# Computational modelling of chromosomally clustering protein domains in bacteria

**DOI:** 10.1186/s12859-021-04512-x

**Published:** 2021-12-14

**Authors:** Chiara E. Cotroneo, Isobel Claire Gormley, Denis C. Shields, Michael Salter-Townshend

**Affiliations:** 1 School of Medicine, University College Dublin, Dublin, Ireland; 2 Conway Institute of Biomolecular and Biomedical Research, University College Dublin, Dublin, Ireland; 3 School of Mathematics and Statistics, University College Dublin, Dublin, Ireland

**Keywords:** Bacteria, Evolution, Genes, Gene function, Domains

## Abstract

**Background:**

In bacteria, genes with related functions—such as those involved in the metabolism of the same compound or in infection processes—are often physically close on the genome and form groups called clusters. The enrichment of such clusters over various distantly related bacteria can be used to predict the roles of genes of unknown function that cluster with characterised genes. There is no obvious rule to define a cluster, given their variability in size and intergenic distances, and the definition of what comprises a “gene”, since genes can gain and lose domains over time. Protein domains can cluster within a gene, or in adjacent genes of related function, and in both cases these are chromosomally clustered. Here, we model the distances between pairs of protein domain coding regions across a wide range of bacteria and archaea via a probabilistic two component mixture model, without imposing arbitrary thresholds in terms of gene numbers or distances.

**Results:**

We trained our model using matched gene ontology terms to label functionally related pairs and assess the stability of the parameters of the model across 14,178 archaeal and bacterial strains. We found that the parameters of our mixture model are remarkably stable across bacteria and archaea, except for endosymbionts and obligate intracellular pathogens. Obligate pathogens have smaller genomes, and although they vary, on average do not show noticeably different clustering distances; the main difference in the parameter estimates is that a far greater proportion of the genes sharing ontology terms are clustered. This may reflect that these genomes are enriched for complexes encoded by clustered core housekeeping genes, as a proportion of the total genes. Given the overall stability of the parameter estimates, we then used the mean parameter estimates across the entire dataset to investigate which gene ontology terms are most frequently associated with clustered genes.

**Conclusions:**

Given the stability of the mixture model across species, it may be used to predict bacterial gene clusters that are shared across multiple species, in addition to giving insights into the evolutionary pressures on the chromosomal locations of genes in different species.

**Supplementary Information:**

The online version contains supplementary material available at 10.1186/s12859-021-04512-x.

## Background

Bacterial and archaeal genomes have a characteristic structural organisation where functionally interacting genes, such as those encoding for the subunits of the same protein complex or involved in the same pathway, physically aggregate in blocks (clusters) on the chromosome. This feature provides an evolutionarily advantage by enabling these cells to regulate and trade these genes as a unit, rather than as a set of independent entities [1]. Indeed, contiguous genes on the same DNA strand can be co-transcribed in response to the same stimuli through a shared promoter, as happens within the family of gene clusters known as *operons*, first described in [2]. Adjacent genes are also transferable to other microorganisms within an individual fragment of DNA of relatively moderate size, thus allowing cell-to-cell transmission of whole pathways through a single exchange of genetic material [3, 4].

Microbial gene clusters have been found to be implicated in a wide variety of biological processes that range from basic cell survival, as in the case of clusters of genes encoding for ribosomal subunits [5] or for proteins conferring immunity to bacteriophages’ attacks [6], to highly specialised metabolic pathways that provide, for instance, photosynthetic [7] or magnetotactic [8] abilities. Bacterial clusters are also of pivotal importance in bacterial-induced disease, as observed in *Escherichia coli* strains that gain the ability to infect humans upon acquisition of DNA segments encoding for enterotoxins and other infection machineries [9].

The existence of chromosomal clustering is also relevant in the field of computational biology, in particular for the prediction of the function of bacterial and archaeal genes [10]. Microbial genome sequences can in fact be examined to identify the clustering partners of genes with unknown cellular role and assign them hypothetical functions based on the known properties of the other members of the cluster. For instance, a transporter protein clustering with enzymes catalysing a certain metabolic pathway could be responsible for the import of the substrate of this pathway; coding sequences located close to toxin injection systems could represent unidentified microbial toxins. Computational methods aimed at predicting functional partners, such as [11] often aggregate across a number of common data sources available for many species, such as scientific literature co-mentions of genes, experimental data, inference through homology, as well as gene clustering. However, since gene clustering is the information source available across all bacteria with sequenced genomes, it is important to be able to understand its dynamics and evaluate its predictive power independent of more variable information sources. Ideally gene clustering should rely on more than genomic distances, since operon co-membership and chromosomal 3D conformation (such as Hi-C) could contribute very functionally relevant information. However, since such experimental data is only available for a small subset of genomes, and computational inferences of these properties may introduce biases relating to sequence composition of species, we chose here to focus exclusively on genomic distance among protein domains as the central information to model.

Different approaches to the problem of chromosomal clustering prediction have been proposed in the past, and all share fundamental methodological challenges. One of these challenges comes from the lack of systematic information about the values of the structural properties of clusters of genes, namely their expected total size and average distance (also called intergenic gap) between adjacent clustering genes. A previous study showed that, in *Escherichia coli*, adjacent genes from the same operon tend to have slightly smaller intergenic distances than adjacent genes that are not co-transcribed [12]. Similar properties have been found to apply to the Gram positive model bacterium *Bacillus subtilis* [13]. To our knowledge, no other similar studies have been performed on multiple microorganisms, mostly due to the scarcity of well-annotated datasets of experimentally validated clusters. Clustering parameters are therefore usually either set to arbitrary thresholds [11, 14–23] or inferred from a few well-known clusters from model species [12, 24]. These simplifications, however, are hard to evaluate and it is possible that they may not be equally suitable for all typologies of microbes or clusters.

Here, we illustrate the results of a large-scale analysis of putative chromosomal clusters carried out on publicly available whole-genome sequences from 14,178 archaeal and bacterial strains. This extensive dataset covered microorganisms from a multitude of taxonomical groups, growth environments and metabolisms. Due to the technical and computational challenges related to the processing of such a large amount of genomic data, this represents one of the biggest surveys of chromosomal clustering in bacteria and archaea available to date. As previously proposed by [19], we chose to focus on clusters of protein domain coding regions instead of whole genes. Indeed, proteins, and consequently protein coding genes, have been widely shown to have a modular structure consisting of one or more regions with (semi-)autonomous function and structure. These regions, called *protein domains*, can either exist as independent proteins or aggregate in different combinations that yield different protein products. It has been extensively argued that protein domains are the true functional and evolutionary units of protein coding genes [25], and it is thus reasonable to also treat them as units of chromosomal clustering.

All the genome assemblies in the dataset were examined for already known clusters of protein domain coding regions by the design and application of a novel method. This approach, based on the concept that the products of clustering genes have related roles within the cell, reduces the need for arbitrary parameter choices in clustering prediction models. We show that selecting for regions encoding for protein domains with shared functional annotations, mainly assigned based on their sequence similarity with experimentally characterised domains, leads to an enrichment of regions located proximally to each other. This proximity can be modelled using probability distribution functions that associate the distance on the chromosome of two domains to their probability of being part of the same cluster. The parameters of these distributions seem not to depend on the functional classes of the assayed domains or on the biological features and chromosomal structures of single microorganisms, and could be applied to the computational prediction of novel clusters.

## Results

**Fig. 1:**
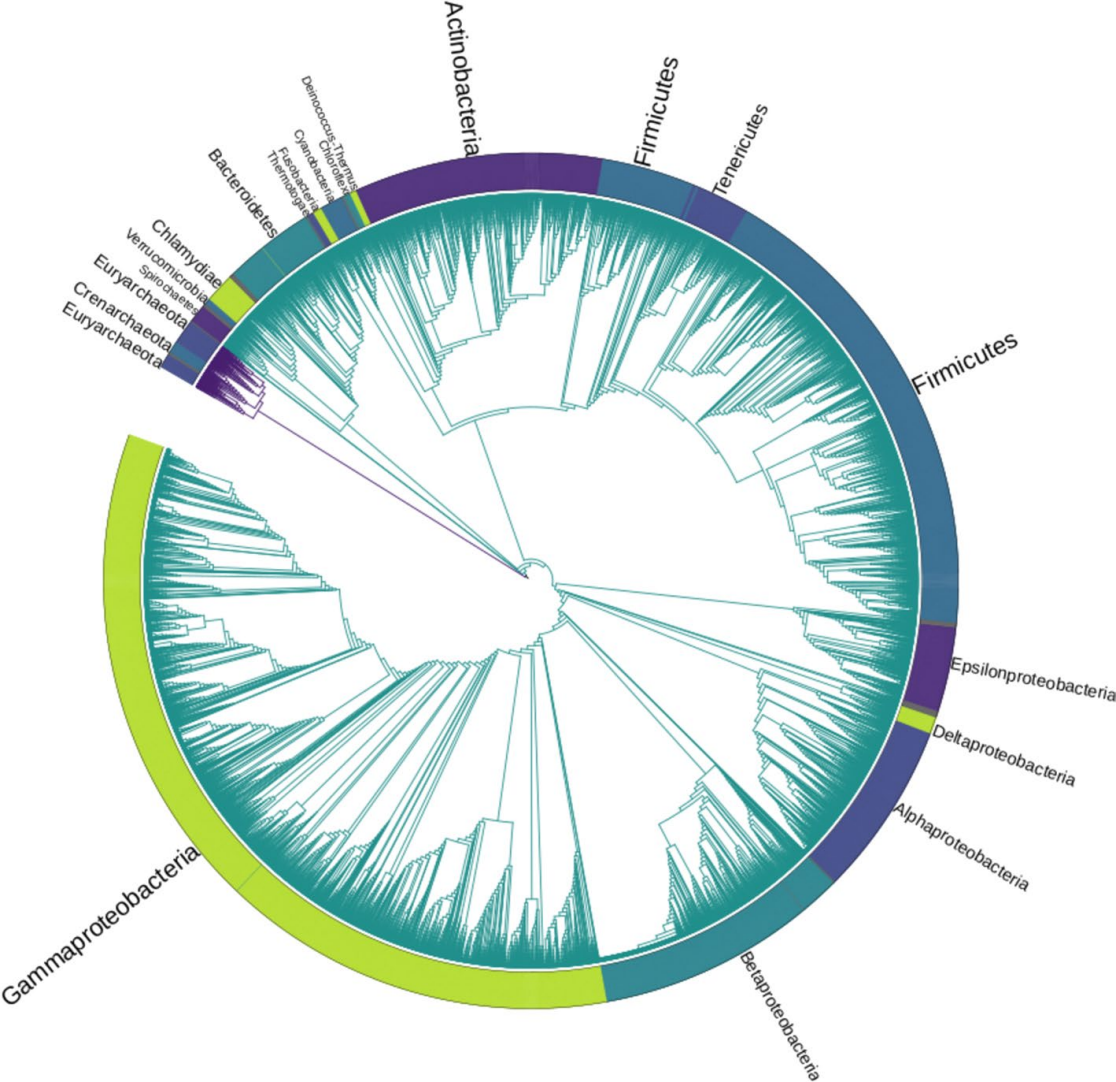
Maximum likelihood phylogenetic tree of the 14,178 complete genome assemblies examined in this analysis. The tree was obtained from a concatenated alignment of the sequences of the 107 housekeeping protein coding genes listed in Additional file 1: Table S1. Purple tree branches correspond to archaea, while the remaining branches correspond to bacteria. Only the main phylogenetic groups, composed of more than 20 assemblies each, are annotated. The Tenericutes clade is branching from within Firmicutes in accordance with the current established view on the origin of this group [26, 27]

This study was conducted on a dataset of 14,178 genome assemblies, corresponding to 13,883 bacterial and 295 archaeal strains. Assayed bacterial assemblies included representatives for 38 unique phyla (with 4 assemblies not assigned to any known phyla), 1138 genera (113 assemblies with unassigned genus) and 4180 species. Of these species, 873 were represented by more than one strain, up to a maximum of 839 strains for *Escherichia coli*. Archaeal assemblies contained strains from 5 unique phyla (1 unassigned), 101 genera (5 unassigned) and 228 species. Only 26 archaeal species had more than one strain representative, with *Saccharolobus solfataricus* having the maximum number of strains (12). A phylogenetic tree showing the taxonomy of all the strains used in this analysis is shown in Fig. 1.

**Fig. 2:**
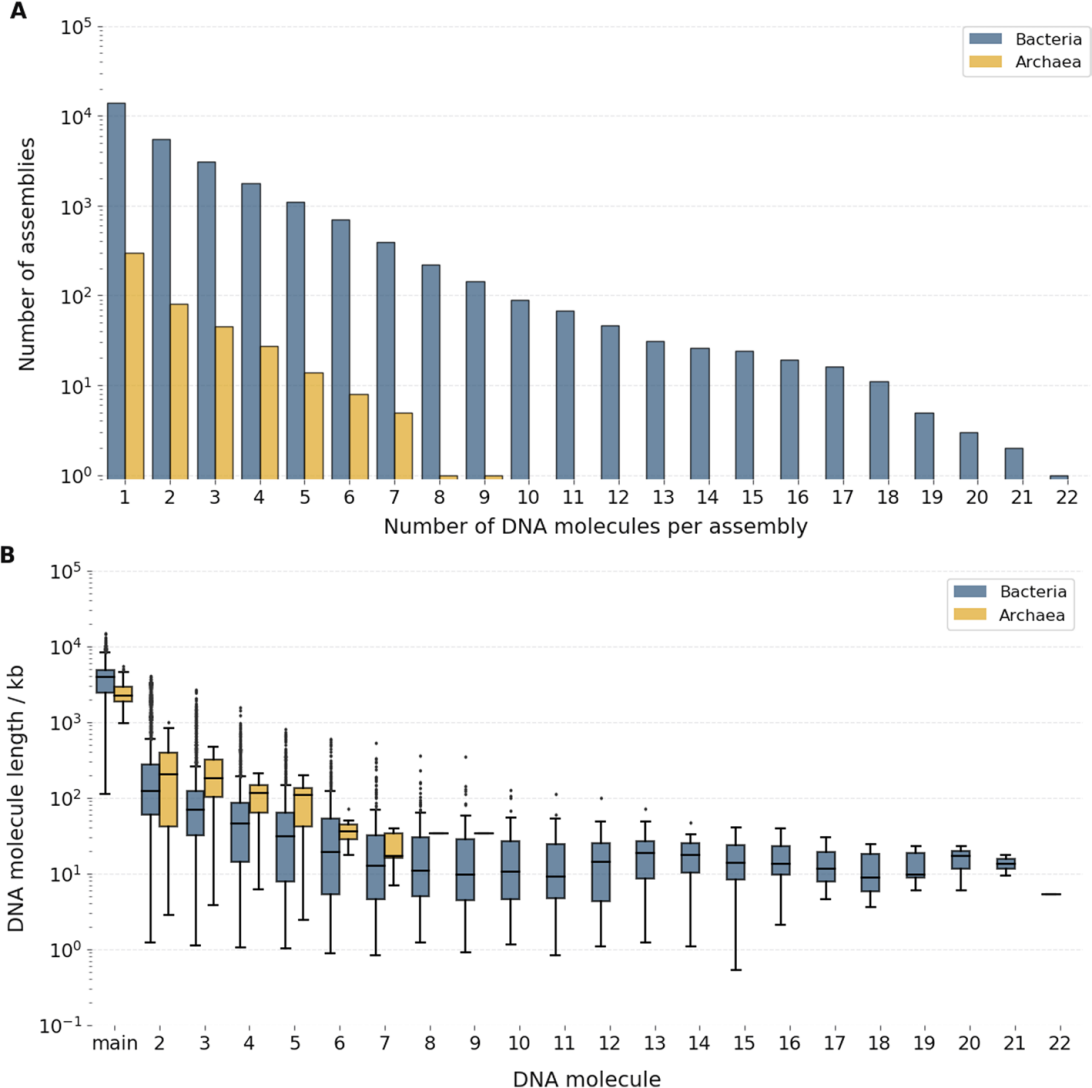
Properties of the DNA replicons found in the 14,178 analysed genome assemblies. Panel **A** shows the number of DNA replicons in the 295 archaeal and 13,883 bacterial genome assemblies analysed; panel **B** shows the distribution of DNA replicon sizes in the same dataset

The genome of most known archaea and bacteria consists of one or more independent DNA elements capable of self-replication (*replicons*). It is usually possible to recognise a main, longer DNA molecule (chromosome), often of circular shape, accompanied by one or more shorter replicons. In this dataset, 8,337 (60.05%) of the total bacterial and 214 (72.54%) of the total archaeal assemblies consisted of a single chromosome, with the remaining genomes containing between two and 22 (bacteria) or 9 (archaea) replicons each (Fig. 2, panel A). The length of the main chromosome in bacteria ranged from the 112 kb of Candidatus *Nasuia deltocephalinicola* to the 14.782 Mb of the soil bacterium *Sorangium cellulosum* So0157-2, with a median length of 3.925 Mb. In archaea, it ranged from 952 kb (candidate strain of *Mancarchaeum acidiphilum*, *Candidatus Micrarchaeota*) to 5.440 Mb (*Haloterrigena turkmenica* DSM 5511), with a median of 2.226 Mb. Additional replicons were usually shorter (Fig. 2, panel B), with a median length of 66 kb for bacteria and 129 kb for archaea.

**Fig. 3:**
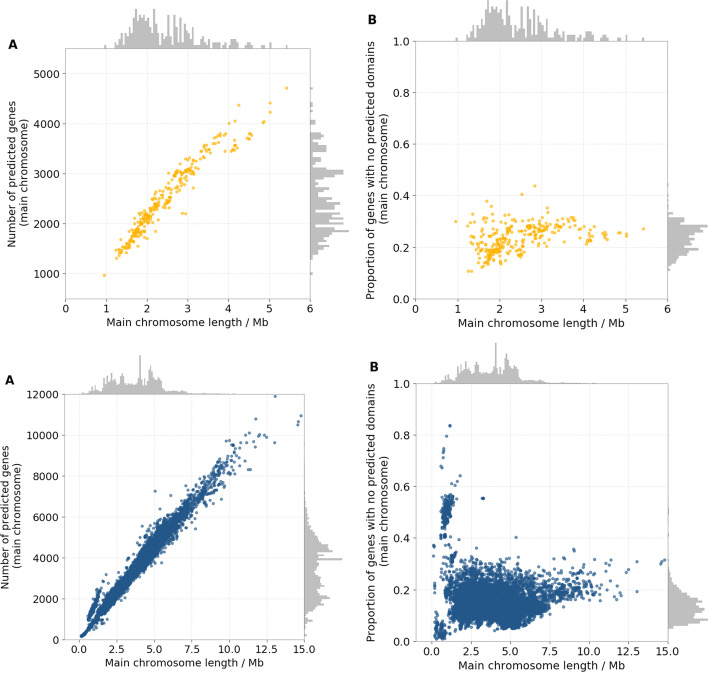
Relationship between the length of the main chromosome and the number of annotated genes or number of genes without predicted protein domain coding regions in the 14,178 genome assemblies analysed. Panels **A** show the total number of annotated gene coding sequences versus the length of the main chromosome in the 295 archaeal (top row) and 13,883 bacterial (bottom row) genome assemblies. Panels **B** show the number of annotated genes containing no predicted Pfam protein domain coding regions on the main chromosome versus the length of the main chromosome in the 295 archaeal (top row) and 13,883 bacterial (bottom row) genome assemblies

Our analysis considered protein domain coding regions as fundamental units of chromosomal clustering in place of whole genes (see  Background and [25] for justification of this choice). Accordingly, predicted gene sequences from every assembly were segmented into one or more sub-sequences, corresponding to protein domain coding portions (hereby referred to as “*domains*”). We used the online database Pfam (https://pfam.xfam.org/, [28]) to annotate domains.^1^ Currently on its 32nd release, Pfam contains information regarding 17, 929 families of protein domains found in eukaryotes, bacteria or archaea. Distinct domains found within the same gene coding sequence were labelled as *fused*. More than one copy (homolog) of the same domain was sometimes found within the same chromosome or gene. The median percentage of predicted genes containing no matches for any Pfam protein domain families was 12.9% in bacteria and 24.24% in archaea (Fig. 3). In total, 14,662 different Pfam domain families were predicted to be present at least once in the dataset.

**Fig. 4:**
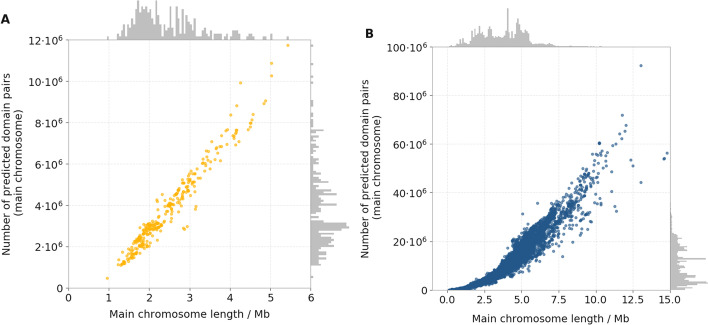
Relationship between main chromosome size and number of pairs of predicted Pfam coding regions in the 14,178 assemblies analysed. Panel **A** shows the number of predicted pairs of protein domain regions with unique coordinates versus the length of the main chromosome in the 295 archaeal genome assemblies. Panel **B** shows the same variables for the 13,883 bacterial genome assemblies

### Pre-existing functional annotations can be used to model known clustering domains

We first reviewed putative clustering domains present in any of the 14,178 total genome assemblies to obtain information about the typical expected structural properties of clusters. Every assembly was tested individually to verify whether these properties were strain-specific, depending for instance on the size of the genome or habitat of each organism, or if they followed some constant patterns.

It is straight-forward to see how, under no previous assumptions about the structure of microbial chromosomal clusters, the minimum amount of domains required to form a cluster is two. Accordingly, our analysis started with the definition of all the possible pairing of domains found on the main chromosome of each assembly. For instance, for a chromosome containing four domains $a_1, a_2, b, c$ (with $a_1$ and $a_2$ being two copies of domain *a* located in different regions of the chromosome) the complete set of these pairs was $\{$(domain $a_1$, domain *b*), (domain $a_1$, domain *c*), (domain $a_2$, domain *b*), (domain $a_2$, domain *c*), (domain *b*, domain *c*), (domain $a_1$, domain $a_2$)$\}$. Pairs involving copies of the same domain, such as (domain $a_1$, domain $a_2$), were excluded from further analyses. In bacteria, the total number of domain pairs per chromosome ranged between approximately 11 thousand (strain PUNC of Candidatus *Nasuia deltocephalinicola*) to just over 92 million (Gram positive *Nonomuraea* sp. ATCC 55076), with a median of just over 9 million. In archaea, the same variable went from just under 464 thousand (Candidatus *Mancarchaeum acidiphilum* strain Mia14) to approximately 11.8 million (*Saccharolobus solfataricus* strain SULG), with a median approximately 3 million (Fig. 4).

Our next step was to examine the arrangement on the chromosome of those pairs where the two domains had similar known cellular roles, which were the best candidates to be part of the same cluster. These pairs were identified by annotating every domain in the dataset with Gene Ontology Biological Process (GOBP) terms, a set of standardised labels that describe previous knowledge about the involvement of protein domains in specific cellular pathways. For instance, domains corresponding to subunits of the ribosome were linked to the GOBP term *translation* (GO:0006412); domains found in bacterial flagella were annotated with *bacterial-type flagellum dependent cell motility* (GO:0071973). Other domains corresponding to known toxins or other infection factors were annotated with the more generic process *pathogenesis* (GO:0009405). All domain pairs on each chromosome were then divided into two subsets: (1) *functionally interacting pairs*, when the two involved domains had at least one identical GOBP annotation, and (2) *other pairs*, when the two domains did not share any GOBP annotations (including cases when one or both domains had no associated GOBP annotations at all). Note that, out of the 14,662 types of domains found at least once in the dataset, 11,947 (81.45%) were not associated to any GOBP term, meaning that the function of most of these domains is still unknown or poorly annotated in public databases.

**Fig. 5:**
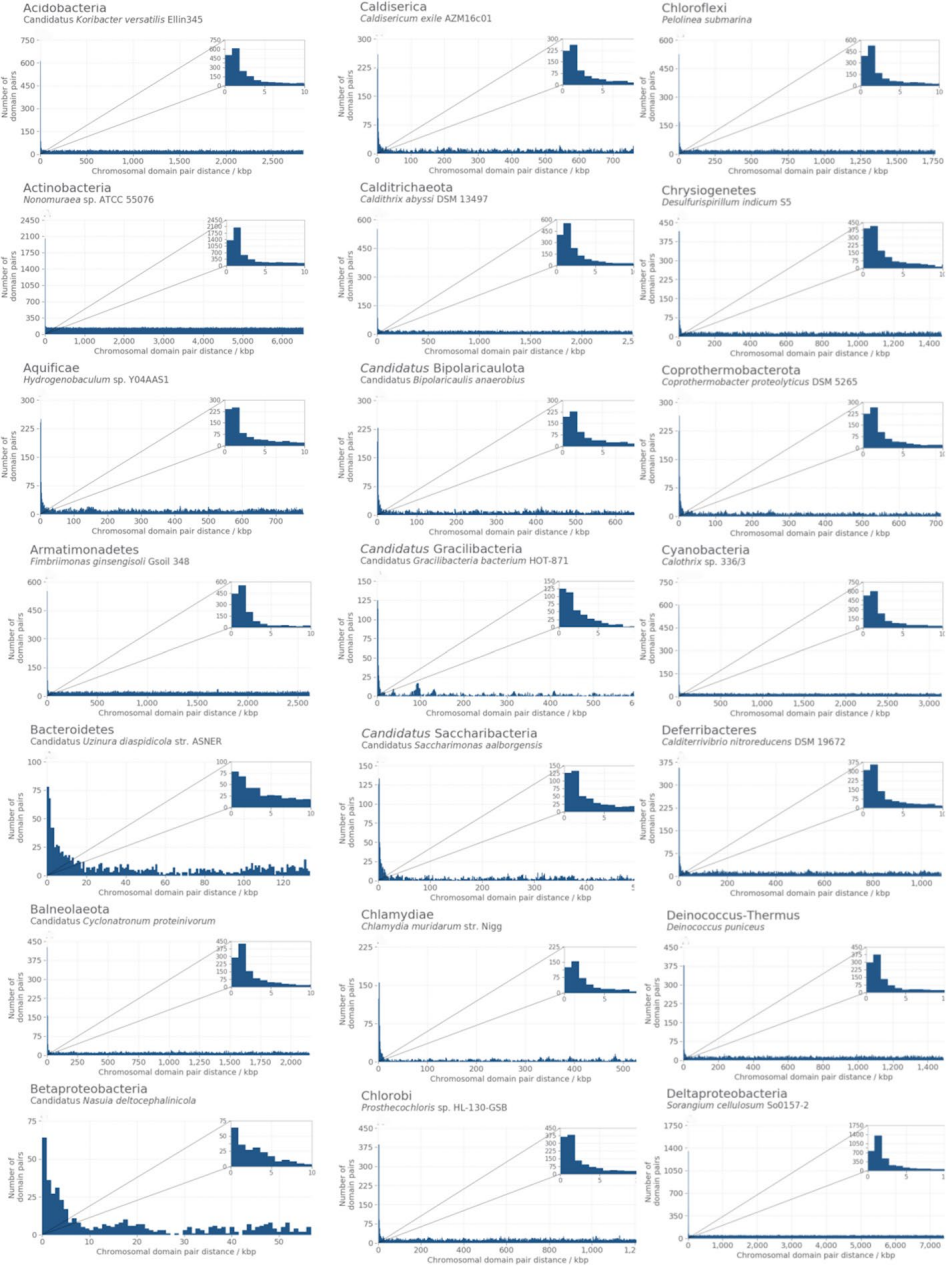

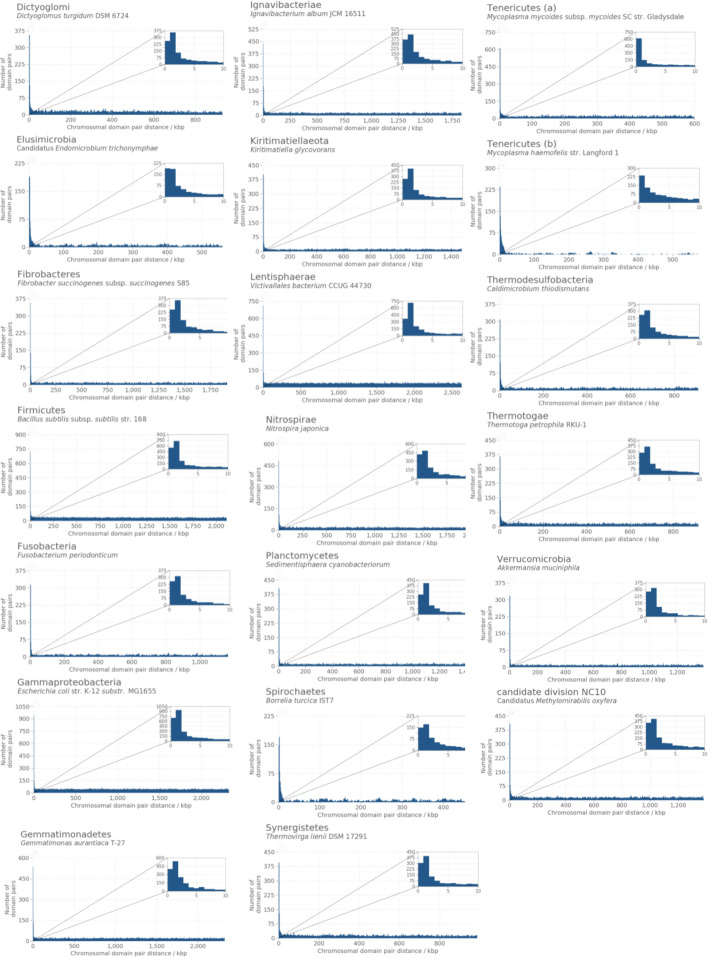
Distributions of chromosomal distances for pairs of Pfam protein domain coding regions in different bacterial phyla. Chromosomal distance, expressed in number of base pairs, between the two members of all pairs of domains where the two domains had one or more Gene Ontology Biological Process (GOBP) annotation in common (*functionally interacting pairs*). Inset on top right of each panel is a zoomed in view of the first 0 to 10 kbp to highlight the peak observed at low distances. Additional file 1: Fig. S1 includes these figures along with the same distribution for pairs where the two domains did not have any common GOBP annotations

**Fig. 6:**
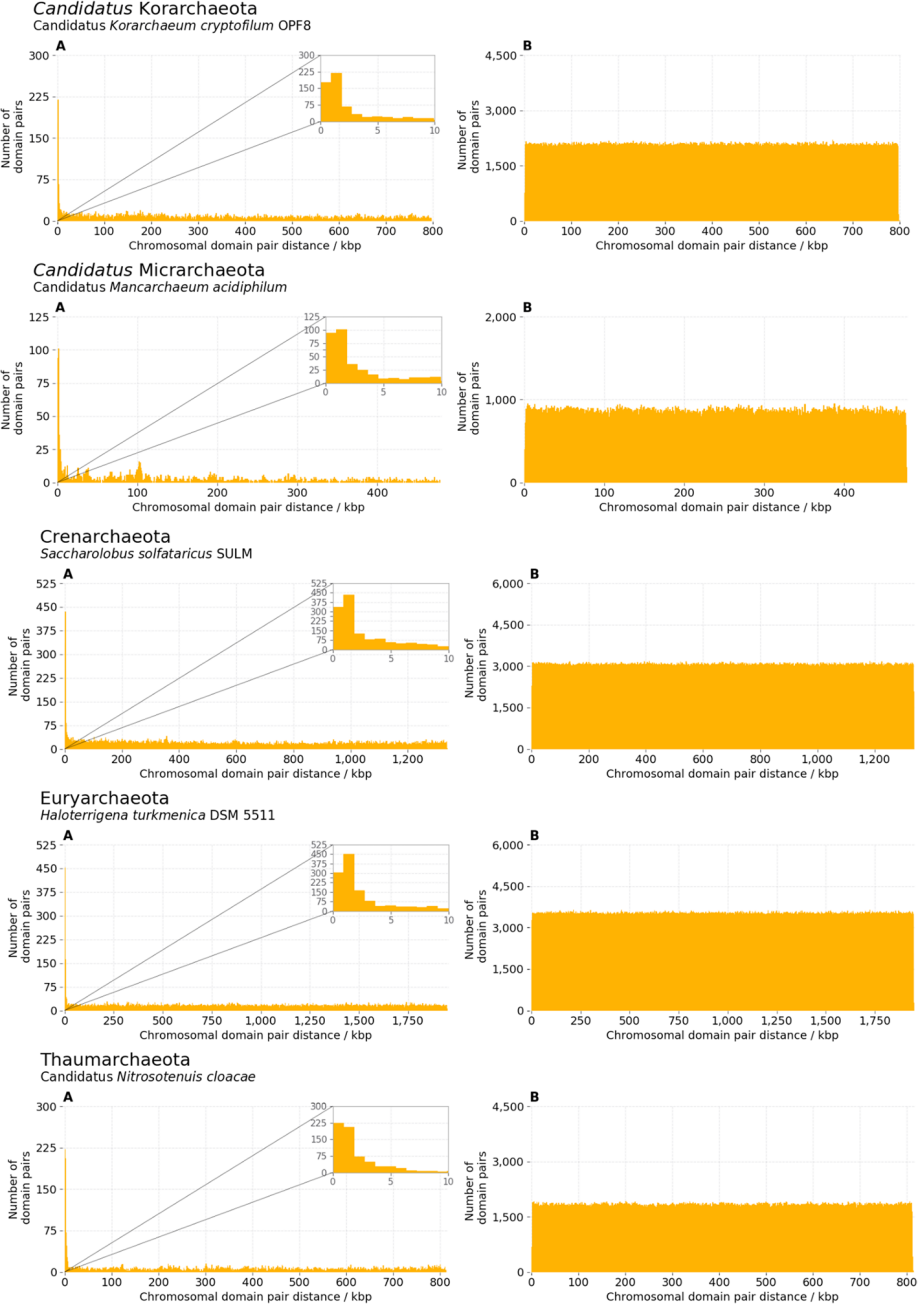
Distributions of chromosomal distances for pairs of Pfam protein domain coding regions in different archaeal phyla present in the dataset. Panels **A**: chromosomal distance, expressed in number of base pairs, between the two members of all pairs of domains where the two domains had one or more Gene Ontology Biological Process (GOBP) annotation in common (*functionally interacting pairs*). Panels **B**: same distribution for pairs where the two domains did not have any common GOBP annotations (*other pairs*)

The position of the two members of the above defined functionally interacting pairs on each chromosome was assessed by measuring the size of the smallest DNA fragment having the two members of the pair at the two ends (*pair chromosomal distance*). In all 14,178 genome assemblies, these distances were approximately uniformly distributed between zero and half the total length of each chromosome, with the exception of a notable peak of observations in correspondence of the beginning of this distribution (shown in Figs. 5 and 6 (panels A) for a select subset of strains). This peak, corresponding to domains located roughly within 5 kb from each other, was consistently observed in all examined chromosomes. To test whether the peak of closely mapping pairs was characteristic of functionally interacting domains, the same analysis was repeated on domain pairs with no shared GOBP annotations. In this case, all measured chromosomal distances were uniformly distributed between zero and half the length of the chromosome, with no detectable enrichment of domains mapping close on the chromosome (Figs. 5 and 6, panels B). This behaviour was consistent in all genome assemblies, with the exception of some *Mycoplasma* strains such as *Mycoplasma haemofelis* str. Langford 1 (Fig. 5, panel Tenericutes (b)), where an enrichment of close pairs was present even for non-functionally interacting domains.

The observed chromosomal distances for pairs of domains indicate that functionally interacting domains display a preference towards being located within a relatively small distance of each other, as expected because of their known tendency to aggregate in chromosomal clusters. Domains with no known functional interactions, on the other hand, appeared to be as likely to be adjacent as on opposite regions of the chromosome (note that, on a circular chromosome, this corresponds to half the total length of the DNA molecule), consistent with previous knowledge that most genes in archaea and bacteria have very close neighbours [17]. The observation of the same uniform distribution of distances for the majority of the functionally interacting pairs also suggested that not all functionally interacting domains (at least, when GOBP annotations are used to define functional interactions) are part of a single chromosomal cluster. These observations were used to build a clustering model as detailed in Methods, and the parameters of the models were then estimated in each assembly to compare their values across different organisms.

### Clustering parameters are stable across microbial strains, with the exception of intracellular parasites and endosymbionts

**Fig. 7:**
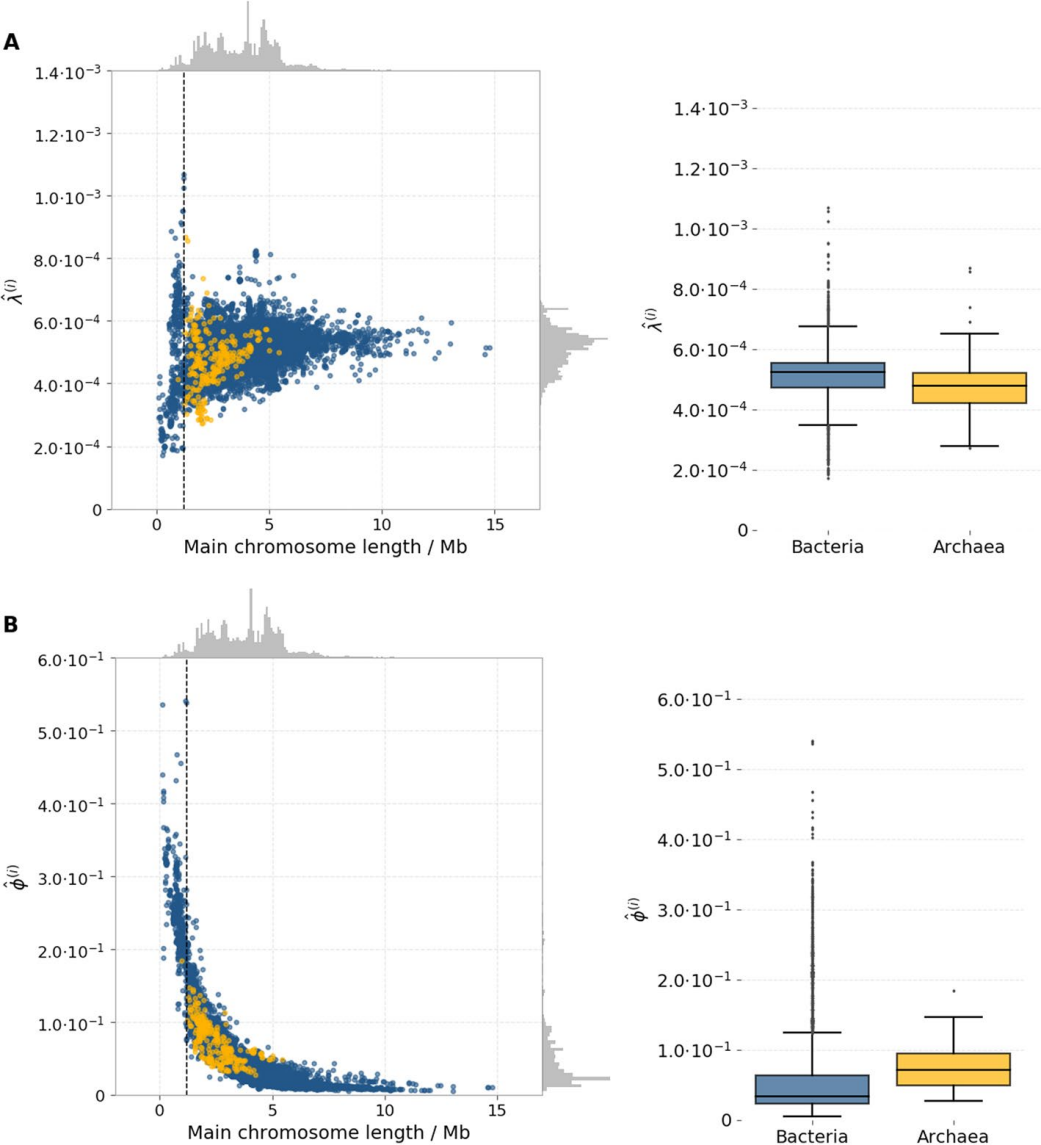
Assembly-specific estimates of clustering parameters obtained in each of the 14,178 genome assemblies analysed. The plots show estimate value for the model’s clustering parameters obtained after fitting the clustering model on chromosomal distances measured for functionally related pairs on each of the 14,178 genome assemblies individually. Panel **A**: estimated values of the rate parameter of the exponential distribution characteristic of clustering pairs of domains (${\hat{\lambda }}^{(i)}$); panel **B**: expected proportion of clustering pairs out of the total functionally related pairs (${\hat{\phi }}^{(i)}$). The vertical dashed line separates organisms with more variable estimates from the rest

The fitting of the clustering model on functionally related pairs in each assembly rendered 14,178 assembly-specific estimates for the two parameters $\lambda ^{(i)}$ and $\phi ^{(i)}$ (Fig. 7). For $\lambda ^{(i)}$, representing the rate parameter of the distribution of distances of clustering pairs, these estimates ranged between a minimum of 1.70 $\times$ 10$^{-4}$, in Candidatus *Uzinura diaspidicola* str. ASNER (whose chromosomal pair distances are shown in panel Bacteroidetes, Fig. 5), to 1.07 $\times$ 10$^{-3}$ for *Mycoplasma mycoides* subsp. *mycoides* SC str. Gladysdale (panel Tenericutes (a) in Fig. 5). The mean and variance of the exponential distribution are the reciprocals of the rate parameter and of its square, respectively. Accordingly, the above values of ${\hat{\lambda }}^{(i)}$ corresponded to an expected chromosomal distance of 5,882.35 base pairs in *U. disapidicola* ASNER (which has an average protein coding region length of 913.34 bp and an average intergenic spacer length of 153.36 bp) and 934.58 bp. in *M. mycoides* Gladysdale (average protein coding region length: 350.54 bp; average intergenic region length: 243.0 bp).

The estimated values for ${\hat{\lambda }}^{(i)}$ were relatively stable across genome assemblies, with the highest variation observed for genome assemblies where the main chromosome was between 112 kb and 1.193 Mb in length (those to the left of the vertical dashed lines in both panels of Fig. 7). For this subset of genome assemblies, the mean and standard deviation of the estimated rate parameter corresponded to 4.62 $\times$ 10$^{-4}$ and 8.99 $\times$ 10$^{-4}$, respectively (the expected domain chromosomal distance for the mean value of ${\hat{\lambda }}^{(i)}$ was 2,164.50). In the remaining genome assemblies, the mean of ${\hat{\lambda }}^{(i)}$ was 5.16 $\times$ 10$^{-4}$, with a standard deviation of 6.25 $\times$ 10$^{-4}$ (expected chromosomal distance for the mean value of ${\hat{\lambda }}^{(i)}$ : 1,937.98). No appreciable differences were observed for estimates computed on archaeal versus bacterial genome assemblies. The set of organisms with short chromosomes and highly variable ${\hat{\lambda }}^{(i)}$ values consisted of strains from the genera *Blattabacteria*, *Borreliae*, *Chlamidiae*, *Entoplasmae*, *Mesoplasmae*, *Mycoplasmae*, *Rickettsiae*, *Spiroplasmae*, *Ureaplasmae* and other less frequent species, all characterised by an obligated endosymbiontic or intracellular-parasitic lifestyle.

Values of ${\hat{\phi }}^{(i)}$, the proportion of clustered pairs out of all pairs, are larger for very small genomes (Fig. 7B). The smallest observed ${\hat{\phi }}^{(i)}$ was 4.7 $\times$ 10$^{-3}$ (*Nonomuraea* sp. ATCC 55076; panel Actinobacteria in Fig. 5); meaning that 47 of every 10,000 pairs of functionally related domains in this organism are expected to cluster together. The maximum observed ${\hat{\phi }}^{(i)}$ was 5.403 $\times$ 10$^{-1}$, obtained in *Mycoplasma haemofelis* str. Langford 1 (Fig. 5, panel Tenericutes (b)), that corresponds to an expected value of 5,403 clustering pairs for every 10,000 functionally related pairs. As in the case of the estimates for the rate parameter, a high min-max variation in ${\hat{\phi }}^{(i)}$ was again observed for strains with relatively short chromosomes. Organisms with chromosomal length between 112 kb and 1.193 Mb had values of ${\hat{\phi }}^{(i)}$ ranging between 7.46 $\times$ 10$^{-2}$ and 5.403 $\times$ 10$^{-1}$, with a mean of 2.33 $\times$ 10$^{-1}$ (2,330 clustering pairs every 10,000). In the rest of the dataset, the mean ${\hat{\phi }}^{(i)}$ value was 4.33 $\times$ 10$^{-2}$ (433 clustering pairs every 10,000). This overall pattern may reflect that these smaller genomes are enriched for clustered housekeeping gene complexes—when taken as a proportion of the total genes—with a corresponding reduction in non-essential genes that may be less tightly clustered.

**Fig. 8:**
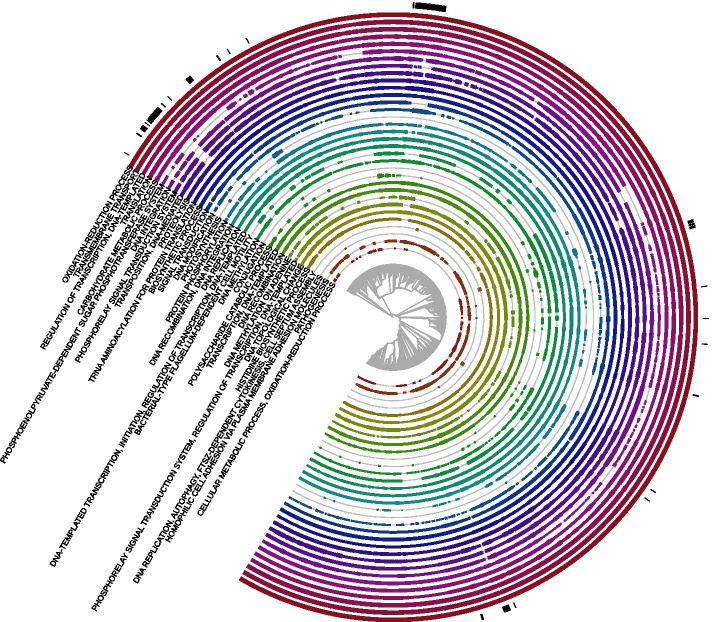
Gene ontology biological process annotations shared by pairs assigned to the clustering pairs sub-population in any of the 14,178 genome assemblies analysed. Pairs assigned to the clustering pairs sub-population during fitting with the Expectation-Maximisation Algorithm were selected as those pairs with estimated ${\hat{z}}_{(a,b)}^{(i)} \ge$ 0.8. For clarity of visualisation, only annotations shared by at least 5% of the total hypothetical clustering pairs from each organism are shown. Black bars on the outermost layer of the plot indicate organisms with chromosomal length between 112 kb and 1.193 Mb

### Hypothetical clustering domains are involved in different cellular processes

After the definition and parameterisation of the model, a further analysis step was taken in order to assess what kind of functionally related domains appeared to be clustering together. As explained more in detail in Methods, the fitting of the model with the Expectation-Maximisation Algorithm returned, for each pair (domains *a* and *b*) on every chromosome *i*, an indicator variable ${\hat{z}}_{(a,b)}^{(i)}$ whose value ranged in the interval [0, 1]. Values of ${\hat{z}}_{(a,b)}^{(i)}$ closer to one indicated that, given the observed chromosomal distance and the model, domains *a* and *b* were likely to be part of the clustering sub-population; here, we considered functionally related domain pairs with a value of ${\hat{z}}_{(a,b)}^{(i)}$ higher than 0.8 as hypothetical clustering pairs.

The hypothetical clustering pairs found across all 14,494 bacterial and archaeal strains shared a total of 1039 different GOBP annotations; the most frequent of these annotations are summarised in Fig. 8. The most ubiquitous shared annotations corresponded to generic, high-level molecular processes such as oxidation-reduction, transmembrane transport, proteolysis, protein phosphorylation, DNA methylation; DNA modification, and DNA topological change. However, the type of clustering domains annotated with these processes differed across genome assemblies, indicating that these clustered pairs did not belong to copies of the same cluster present in most microorganisms, but rather to independent clusters with similar functional annotations. A second group of frequently shared GOBP terms referred to housekeeping pathways indispensable for basic cellular survival, such as DNA replication, DNA transcription, translation and tRNA aminoacylation for protein translation. As opposed to domains from the previous category, in this case the hypothetical clustering domains carrying these annotations were the same in most assayed bacteria and archaea.

Many putative clustering domain pairs were part of metabolic pathways necessary for the production of energy or the synthesis of biological molecules (carbohydrate metabolic process; phosphoenolpyruvate-dependent sugar phosphotransferase system; polysaccharide catabolic process; biosynthetic process; histidine biosynthetic process; photosynthesis). Some of these annotations were widespread among organisms, while others were specifically found just in some subclades, as in the case of pairs involved in photosynthesis, that were found within the *Chlorobi* subclade. Other hypothetical clustering functionally related pairs were involved in regulatory systems used by cells to sense external stimuli and trigger adequate responses (signal transduction; regulation of transcription, DNA templated; phosphorelay signal transduction system). A further category of functional annotations often shared by clustering pairs referred to pathways involved in the horizontal transfer of DNA segments to other organisms (DNA recombination; DNA integration; transposition, DNA mediated; conjugation), in accordance with the knowledge that chromosomal gene clusters are often exchanged between cells. Lastly, some group of microorganisms carried clustering pairs annotated with infection-related functions such as bacterial-type flagellum-dependent cell motility, cell adhesion or pathogenesis. Organisms with shorter chromosomes and atypical clustering parameters did not display specific patterns in the type of functions carried out by their putative clustering domains.

## Discussion

We have defined a probabilistic model describing the structural properties of chromosomal clusters of protein domain-coding regions in 14,178 bacterial and archaeal species. To our knowledge, this is the first time that a dataset of comparable size has been used for the analysis of chromosomal gene clusters. These chromosomal aggregates of functionally related genes are thought to be ubiquitous across microorganisms and to involve a significant portion of their total coding sequences [29]; however, mainly due to experimental challenges in their identification, no information about known domain clusters is available for most microbial genomes. Here, we observed that protein domains already known for being involved in the same cellular pathway have a tendency to be proximal to each other on the chromosome. These sets of proximal, functionally related regions were selected as members of hypothetical clusters of domains, and used to build a probabilistic model that links their positions on the chromosome to their likelihood of being members of the same cluster. The fitting of the model on 14,178 archaeal and bacterial strains showed that the parameters of this clustering model are stable across bacterial lineages, with the exception of a small subset of organisms consisting of obligate intracellular parasites and endosymbionts.

The similarities in clustering parameters did not appear to depend on a shared set of domains forming similar clusters in multiple species, but rather on the existence of fixed structural properties shared by clusters with different functions. Indeed, only some of the identified putative clusters, in particular those involved in basic housekeeping pathways such as cell replication and protein expression, were widespread across species; others, such as clusters conferring photosynthetic or pathogenic abilities, were confined to specific phylogenetic groups inhabiting environments in which these processes are needed for their survival. Additionally, each species carried multiple clusters involved in different types of cellular functions. Multiple hypotheses can be made to try to explain the observed consistencies in clustering parameters. It is possible that, during evolution, most microbial clusters would converge to similar structures because of constraints posed by the molecular processes involved in their expression and transmission. For instance, coding sequences clustering forming operons are often transcribed within a single polycistronic mRNA molecule. It has been observed that bacterial mRNA length can correlate with a decrease in stability and an increase in degradation rate [30, 31]. This may act as a factor that limits the total size of operons (by favouring, for instance, operons made of fewer genes or with smaller intergenic regions), to avoid the production of oversized and therefore unstable transcripts. It is also known that clustering genes can be exchanged across microbial species [3, 32], and sometimes even between microbes and higher eukaryotes [33]. These horizontal gene transfer events take place when a cell collects an exogenous DNA fragment from the environment, by direct contact with another cell or through a bacteriophage acting as a “DNA shuttle” [34]. The successful outcome of these DNA acquisitions, however, depends on the size of the fragment itself, as larger pieces of DNA are, for instance, less likely to fit within the head of a bacteriophage or to be successfully uptaken by DNA recombination machineries for integration into the recipient cell’s genome [4]. Lastly, intuition dictates that the chain of evolutionary events required for independent genes to start interacting and at the same time group together on the chromosome should be less probable for large number of genes, favouring the formation of shorter clusters [1].

Bacterial species with intracellular lifestyles were the only class that displayed detectable variations in their clustering parameters. This class of microorganisms has a peculiar life cycle, where they are never in contact with the external environment but mainly propagate inside the cytoplasm of other cells that provide them with nutrients and protection. This lifestyle is connected to a substantial shrinking of their genome, with most genes being made redundant by the presence of similar pathways in the host cell and undergoing inactivation via pseudogenisation [35–37]. The resulting scarcity in gene coding sequences, coupled with the relatively large non-coding regions made of left-over fragments from inactivated genes, makes the analysis of these genomes more subject to random noise. This property, coupled with the fact that these organisms may be involved in fewer horizontal gene transfer events due to their lack of contact with external DNA sources, could explain the observed fluctuations in their clustering parameters.

The main advantage of using a probabilistic model is that it allows us to test for chromosomal clustering without the need for a fixed threshold in terms of number of involved genes or size on the chromosome, but by expressing them more flexibly in terms of probabilities. However, the approach described in this paper has a series of pitfalls, mainly associated with the inaccuracies or gaps in the annotation of Pfam domains. In particular, 13% of the total predicted genes in bacterial genome assemblies and 24% of those in archaeal genome assemblies did not match any known Pfam domain coding regions, meaning that some coding portions of these genomes were ignored in all subsequent analyses. This issue will likely become less and less relevant as more information about Pfam domains is gathered, allowing the refinement of these annotations. More importantly, it should be noted that analysing chromosomal proximity of some domains in a single bacterial species is, by itself, not sufficient to generate hypotheses about the presence of clusters. The high compactness and gene density of microbial genomes lead most consecutive genes to map very close to one another, even in the absence of functional interactions [17].

The models presented here should prove of utility in statistical investigation of domain clustering to define clustered domain pairs. This could in principle be performed independently, or in combination with machine learning approaches that can draw on other shared features of the pairs. Such features might include G+C content or more detailed sequence features [38] that, for very highly mobile clusters undergoing high levels of horizontal gene transfer, might relate to aspects of their common mutational history across their shared previous hosts.

It is likely that, within each genome, there are groups of genes for which the distance models may differ, such as genes that may be distinguished by particular regulatory/operon structures, or by distinct patterns of lateral gene co-transfer between strains. However, such sub-analyses are tricky, as the definition of such subgroups would need to be large enough in order to have statistical power for parameter inference.

The distance estimates presented here are dependent on the reliability of GO terms. While it is likely that there is error in GO terms, we would have anticipated that such error would be likely to increase with the computational inference of such terms in non-model species. In spite of this, we found no obvious indication that parameter estimates were markedly different for genomes which were more distantly related to model organisms which have been the focus of extensive experimental annotation work. This suggests that the parameter estimates are likely robust to reasonable levels of error in the individual GO terms used in the analysis.

The most obvious application of the models may be towards improving methods for the discovery of gene functional relationships on the basis of chromosomal co-clustering across genomes, such as are incorporated as a strand of evidence into the STRING database of putative functional relationships [11]. The reliability of these estimates will depend on the correct statistical handling of genomic similarity of closely related strains. A straightforward analysis of clustering across all genomes will end up dominated by the clustering of many syntenic genes seen in very closely related species; this would provide little insight into the more informative deeper clustering of functionally related domains that persists over longer evolutionary distances.

## Conclusions

This study represents one of the biggest surveys of chromosomal clustering in bacteria and archaea available to date. We first demonstrated that functionally related pairs of protein domain coding regions are typically found close together on bacterial and archaeal chromosomes compared to pairs of domains that are not functionally related. We then fitted a two component mixture model to the observed intergenic distances between Pfam domains across a large set of bacteria and archaea species. The first component models these distances as being uniformly distributed, corresponding to a neutral evolutionary pressure on location. The second component assumes the existence of an evolutionary pressure to keep protein domains that are functionally related close together on the chromosome and the pairwise distances are modelled as exponentially distributed, with a species-specific rate parameter.

We find that both the relative proportion of each component and the exponential decay rate of our clustering model seem not to depend on the functional classes of the assayed domains or on the biological features and chromosomal structures of single microorganisms, and can be applied to the computational prediction of novel clusters. Finally, our mixture model estimates the probability of belonging to the clustering mixture component for each domain pair; that is, the probability that they are functionally related in that strain. Future work will use our approach and results to discriminate between these two groups in the absence of previous information about their cellular role, and to apply the developed model to the prediction of new putative evolutionarily conserved clusters occurring across multiple strains of bacteria and archaea. This will involve combining the strain specific probabilities while accounting for phylogenetic distance and leverages the main result of this work which is that the mixture model parameters are stable across species. Such an approach could also be used to assess the reliability of GO terms, as pairs that share a term which are found not to have a small intergenic distance conserved across species is indicative of annotation error.

## Methods

### Dataset of archaeal and bacterial genomes

#### Microbial genome assemblies

The study was conducted on a dataset of genome assemblies from 13,883 bacterial and 295 archaeal strains, for a total of 14,178 complete genomes selected (for the list see https://doi.org/10.6084/m9.figshare.15035619.v1), from the NCBI Genbank Genomes database (https://ftp.ncbi.nlm.nih.gov/genomes/genbank/), among the complete genome assemblies available in June 2019 that had also passed RefSeq quality checks. Assembly metadata, such as taxonomical identifiers, sources and assembly statuses, were extracted from the summary file available at

https://ftp.ncbi.nlm.nih.gov/genomes/GENOME_REPORTS/prokaryotes.txt. The complete nucleotide sequences of each assembly (available in fasta format in .fna files) were tested for putative gene coding sequences with the microbial genome annotation tool Prokka v1.12 [39] with default software settings. The only exception was assembly GCA_002761215.1, corresponding to Candidatus *Gracilibacteria bacterium* HOT-871, which follows the alternative version of the genetic code known as translation table 25, where the stop codon UGA encodes for the amino acid glycin. In this case, Prokka was run with the option --gcode 25.

#### Annotation with Pfam domain coding regions

All putative gene sequences identified by Prokka were further examined for the presence of protein domain coding regions using hmmscan v3.1b2 [40], with setting --eval 0.001. Hmmscan was run against the target database Pfam release 32.0 (https://ftp.ebi.ac.uk/pub/databases/Pfam/releases/Pfam32.0/Pfam-A.hmm.gz, [28]), a reference collection of Hidden Markov Model profiles for 17,929 protein domain families. The threshold to define whether a gene contained a significant sequence match for a putative Pfam domain was placed at single domain e-value < 0.001. A further filtering step was implemented to deal with cases where multiple domains from the same Pfam domain clan matched within the same gene sequence. These clans are groups of domain families with shared ancestry that have undergone independent evolution, but still retain high sequence similarity. This means that, in presence of a true match for a member of a clan, other members of the same clan can also align with the same region, albeit with lower scores. Whenever this happened, only the match with the lowest e-value was accepted. Finally, as hmmscan is often not able to determine the exact boundaries of single Pfam domain coding regions within gene sequences, the chromosomal positions of all predicted domains were approximated to the ones of their corresponding gene coding sequences.

#### Identification of known functionally interacting domains

The gene ontology (GO) database [41] is an online resource that provides a series of relationships (annotations) between genes or protein domains and some standard labels (Gene Ontology terms) describing their known molecular or cellular functions. All predicted Pfam protein domains from the 14,178 genome assemblies were annotated with the corresponding Gene Ontology term(s), when available, using the pfam2GO mapping table version 2019/06/01 14:44:40 downloaded from http://geneontology.org/external2go/pfam2go. From these annotations, we selected only those corresponding to Gene Ontology Biological Process (GOBP) terms, using the GO terms description file available at http://purl.obolibrary.org/obo/go.obo. Any two domains found in the same assembly that shared at least one identical GOBP annotation were labelled as functionally interacting.

#### Phylogenetic analysis

A phylogenetic tree describing the evolutionary relationships between the 14,178 examined microbial strains was built from the sequence of 107 protein coding genes (see Additional file 1: Table S1 for a list of housekeeping protein coding genes from archaea and bacteria) previously identified as present in most known bacteria and archaea, such as genes encoding for ribosomal proteins or sub-units of the RNA polymerase [42]. This analysis was carried out using the software wrapper bcgTree v1.0.8 [43]. In detail, the protein sequences of the predicted homologs of these genes in all genome assemblies were identified with hmmscan, concatenated together and aligned with Clustal Omega v1.2.1 [44]. The alignment was refined by selecting only the best aligned blocks using Gblocks v.0.91b [45], and processed to build a Maximum-Likelihood tree with the software FastTreeMP v2.1.8 [46]. The resulting tree was rooted using the archaea subclade as an out-group. The currently accepted taxonomic classification (species, genus, phylum, etc) for each assembly in the dataset was determined by mapping each taxonomic identifier to the current taxonomy of all living beings available on the NCBI database (https://ftp.ncbi.nlm.nih.gov/pub/taxonomy/new_taxdump/new_taxdump.tar.gz).

### Modelling of clustering domains in individual organisms

**Fig. 9:**
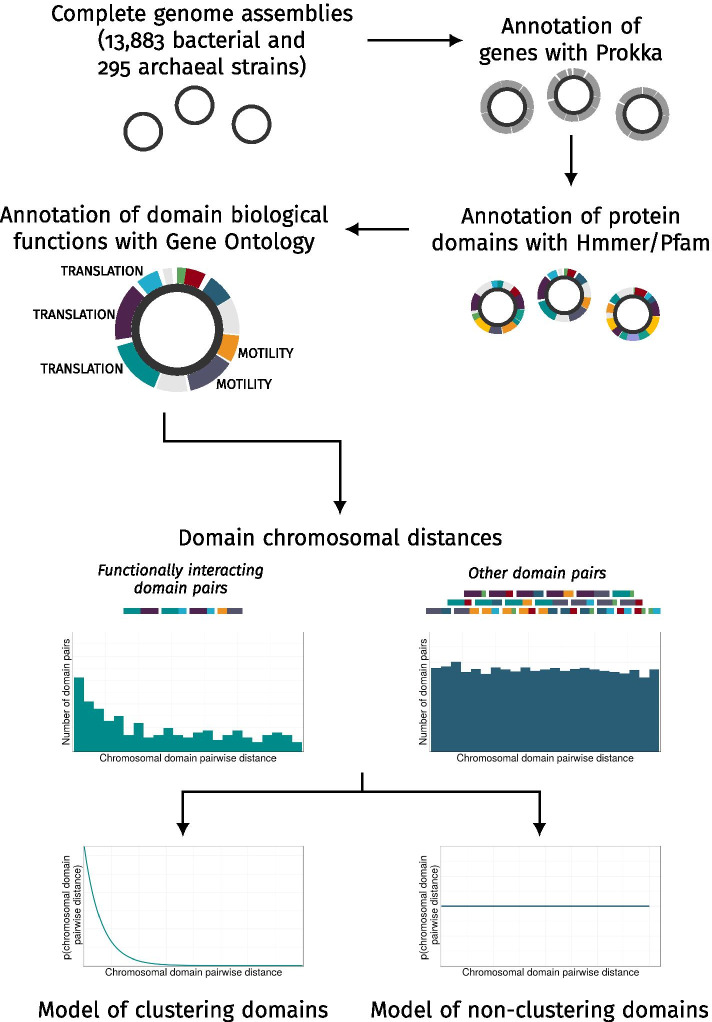
Schematic pipeline of the analyses

An overall visual summary of the complete analysis pipeline is reported in Fig. 9.

#### Chromosomal distances of domain pairs

The probabilistic models of chromosomal distances of clustering domains were obtained by analysing each of the 14,178 genome assemblies independently and only on their main chromosome, which was defined as the longest DNA molecule found in each assembly. The choice to restrict to the main chromosome was made to reduce noise caused by the presence of minor DNA elements such as plasmids, where most domains map very close to each other simply simply because of the small size of the DNA molecule itself. In every chromosome *i*, we measured the chromosomal distance (expressed in number of base pairs) between the two members of all possible pairings (domain *a*, domain *b*) obtainable from the Pfam domains located on the chromosome. This distance (defined in Eq. 1) includes the size of both involved domain coding sequences and of any DNA fragment located between the two.

**Fig. 10:**
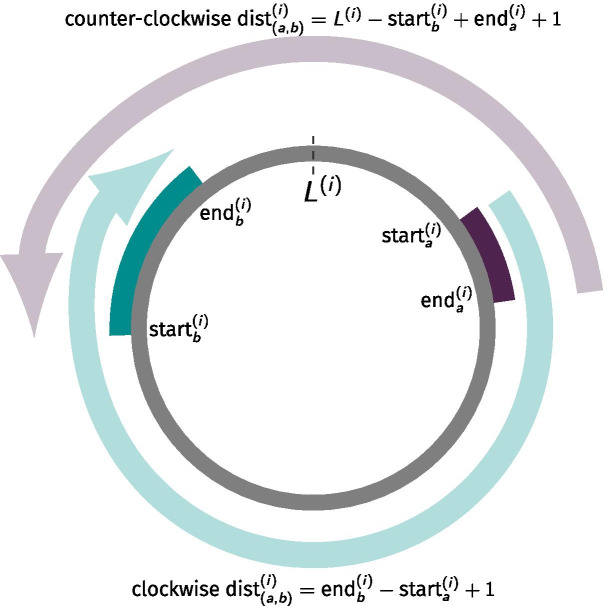
Schematic representation of the method utilised to measure chromosomal distances of pairs of domains. This method was used to measure the chromosomal distance $\text{dist}_{(a,b)}^{(i)}$, expressed in number of base pairs, between two domain coding regions *a* and *b* on a circular chromosome *i* of length $L^{(i)}$. The final value of $\text{dist}_{(a,b)}^{(i)}$ was chosen as the smallest distance measured by travelling the chromosome either clockwise or anti-clockwise

In genome assemblies from bacteria and archaea, gene (and domain) coordinates are conventionally assigned beginning at the origin of replication (located immediately upstream to the predicted homolog of *dnaA* gene) and increasing along the leading strand of the DNA molecule, travelled clockwise, until the origin of replication is reached again. Due to the circular shape of most of these chromosomes, however, domains that appear to be very far according to the values of their coordinates can be very close if the chromosome is examined in an anti-clockwise manner. Accordingly, for each pair (domain *a*, domain *b*) found on chromosome *i*, the corresponding chromosomal distance was defined as the size, expressed in number of base pairs, of the smallest DNA fragment delimited by and including the two domains measured by travelling the chromosome either clockwise or anti-clockwise (as shown in Fig. 10):

1 $$\begin{aligned} \text{dist}_{(a,b)}^{(i)} = \min (\text{end}_{b}^{(i)} - \text{start}_{a}^{(i)} + 1, L^{(i)} - \text{start}_{b}^{(i)} + \text{end}_{a}^{(i)} + 1), \end{aligned}$$

where $\text{start}_{a}^{(i)}$, $\text{end}_{a}^{(i)}$ and $\text{start}_{b}^{(i)}$, $\text{end}_{b}^{(i)}$ are the start and end coordinates of domain *a* and domain *b* on chromosome *i*, respectively, and $L^{(i)}$ is the total length of chromosome *i*, expressed in number of base pairs. These distances are symmetrical, in that $\text{dist}_{(a,b)}^{(i)}$ (clockwise) = $\text{dist}_{(b,a)}^{(i)}$ (counterclockwise); therefore, the order in which the two domains are located on the chromosome is not relevant. Note that, when two domains were located within the same gene, the value of $\text{dist}_{(a,b)}^{(i)}$ corresponded to the size of the gene itself. To avoid over-representation of the same distance due to the presence of multi-domain protein coding genes, all distances corresponding to multiple pairs of domains mapping within the same pair of genes were only counted once.

#### Clustering model based on chromosomal positions

After examining the distributions of chromosomal distances between functionally interacting pairs, we hypothesised these distributions to be the result of the existence of two distinct sub-populations of pairs that can be modelled using two distinct probability distributions. The chromosomal distances of the first sub-population, representing functionally interacting domains grouping together on the chromosome (*clustering pairs*), were modelled using an exponential distribution with rate parameter $\lambda ^{(i)}$:

$$\begin{aligned} \text{Exp}\Big (\text{dist}_{(a,b)}^{(i)} ; \lambda ^{(i)}\Big ) = \lambda ^{(i)} \cdot \exp {\displaystyle \left( -\lambda ^{(i)} \cdot \text{dist}_{(a,b)}^{(i)}\right) } \; \qquad \text{for~dist}_{(a,b)}^{(i)} \ge 0. \end{aligned}$$

Thus clustering pairs are modelled as being likely to be close together on the chromosome with the probability of chromosomal distance between them exponentially decaying with increasing distance. The value of the rate parameter of this distribution was initially considered as characteristic of each chromosome *i* and unknown.

The second sub-population, representing domains that are involved in the same cellular process but whose coding regions are scattered on the chromosome (*non-clustering pairs*), was modelled with a uniform distribution between zero and half of the total length of chromosome *i*:

$$\begin{aligned} \text{U}\bigg (\text{dist}_{(a,b)}^{(i)} ; 0, \frac{L^{(i)}}{2}\bigg ) = \frac{2}{L^{(i)}}\; \qquad \text{for} 0 \le x \le \frac{{L^{(i)}}}{{2}}. \end{aligned}$$

i.e. all chromosomal distances between non-clustering pairs are modelled as being equally probable.

The likelihood of the observed chromosomal distances between the two members of functionally interacting pairs^2^ was then defined using a mixture model consisting of the weighted sum of the these two distributions:

$$\begin{aligned} p(\text{dist}_{(a,b)}^{(i)} \vert a, b \; \text{functionally interacting})& = \phi ^{(i)} \cdot \text{Exp}\Big (\text{dist}_{(a,b)}^{(i)} ; \lambda ^{(i)}\Big ) + \\&(1 - \phi ^{(i)}) \cdot \text{U}\Big (\text{dist}_{(a,b)}^{(i)} ; 0, L^{(i)}/2\Big ), \end{aligned}$$

where the weights $\phi ^{(i)}$ and $(1 - \phi ^{(i)})$ represent the proportion of clustering and non-clustering pairs, respectively, out of the total number of functionally interacting pairs found on chromosome *i*. The allocation of the observed functionally interacting pairs to the clustering or non-clustering population - and consequently the value of $\phi ^{(i)}$—was unknown at this stage.

#### Parameter estimation for the clustering model

The values of the two parameters characterising the above mixture model, namely the rate parameter of the exponential distribution $\lambda ^{(i)}$ and the proportion of clustering pairs over the total functionally interacting pairs $\phi ^{(i)}$, were initially regarded as unknown and characteristic of each chromosome *i*. As these values are not obtainable analytically, their maximum-likelihood estimates were computed with the Expectation-Maximisation (EM) algorithm [47]. This optimisation algorithm is a heuristic procedure commonly employed in statistics to obtain parameter estimates for models with latent properties, that is models where the data display characteristics that can not be directly measured but can be inferred from other variables. In this case, the hidden feature of the data was whether the two members of each functionally interacting pair were part of the same cluster. This property was included in the model by assigning to each pair a binary latent variable $z_{(a,b)}^{(i)}$, that indicated whether the pair was likely to be part of cluster ($z_{(a,b)}^{(i)} = 1$) or not ($z_{(a,b)}^{(i)} = 0$). The values of these indicator variables were initially unknown.

The EM algorithm takes its name by the fact that it computes maximum likelihood estimates by iterating through two steps (Expectation and Maximisation), usually followed by a third step that checks for convergence. In the implementation used in this study, the algorithm started with two initial guesses for the two parameters (called ${\hat{\lambda }}^{(i, 0)}$ and ${\hat{\phi }}^{(i, 0)}$) and updated them by repeating the following three steps for $t =$ 1, 2, $\dots$, up to a maximum of 1,000 iterations:

*(1) Expectation step* Given the most recent estimates for the two parameters of the model, called ${\hat{\lambda }}^{(i, t-1)}$ and ${\hat{\phi }}^{(i, t-1)}$, update the estimates for the latent variables of each pair (domain *a*, domain *b*) on chromosome *i* according to the following equation:

$$\begin{aligned} \hat{z}^{(i, t)}_{(a,b)} = \frac{{\hat{\phi }}^{(i, t-1)} \cdot \text{Exp}\Big (\text{dist}_{(a,b)}^{(i)} ; {\hat{\lambda }}^{(i, t-1)}\Big )}{{\hat{\phi }}^{(i, t-1)} \cdot \text{Exp}\Big (\text{dist}_{(a,b)}^{(i)} ; {\hat{\lambda }}^{(i, t-1)}\Big ) + (1 - {\hat{\phi }}^{(i, t-1)}) \cdot \text{U}\Big (\text{dist}_{(a,b)}^{(i)} ; 0, L^{(i)}/2\Big )}, \end{aligned}$$

where $\text{dist}_{(a,b)}^{(i)}$ is the distance of the two members of the pair on chromosome *i*, obtained according to Eq. 1, and $L^{(i)}$ is the size (in base pairs) of the same chromosome. This is the expected value of the indicator variable of whether the pair are clustering or non-clustering, given the current estimates of the model parameters.

*(1) Maximisation step* Compute new values ${\hat{\lambda }}^{(i, t)}, {\hat{\phi }}^{(i, t)}$ that maximise the expected complete data log-likelihood given the current estimates of the latent variables, that can be written as:

$$\begin{aligned} \begin{aligned} Q\big (\lambda ^{(i)}, \phi ^{(i)} \mid {\hat{\lambda }}^{(i, t-1)}, {\hat{\phi }}^{(i, t-1)}\big )&= \sum _{a \ne b}{\Big (\hat{z}^{(i, t)}_{(a,b)} \cdot \log \Big (\text{Exp}\Big (\text{dist}_{(a,b)}^{(i)} ; \lambda ^{(i)}\Big )\Big )\Big )} \\&\qquad + \sum _{a \ne b} {\Big (\Big (1 - \hat{z}^{(i, t)}_{(a,b)}\Big ) \cdot \log \Big (\text{U}\Big (\text{dist}_{(a,b)}^{(i)} ; 0, {L^{(i)}}/{2}\Big )\Big )\Big )}. \end{aligned} \end{aligned}$$

In detail, the value of ${\hat{\lambda }}^{(i, t)}$ that maximises the above complete data log-likelihood function is obtained by taking the partial derivative of the above function with respect to $\lambda ^{(i)}$ and setting it equal to zero, yielding:

$$\begin{aligned} {\hat{\lambda }}^{(i, t)} = \frac{\sum _{a \ne b}{\hat{z}^{(i, t)}_{(a,b)}}}{\sum _{a \ne b}{\Big (\hat{z}^{(i, t)}_{(a,b)} \cdot \text{dist}_{(a,b)}^{(i)}}\Big )}. \end{aligned}$$

The update estimate for ${\hat{\phi }}^{(t)}$ is then obtained by maximising the complete data log-likelihood with respect to $\phi ^{(i)}$, which results in the computation of the mean of the new estimates of all the latent variables obtained in the estimation step:

$$\begin{aligned} {\hat{\phi }}^{(i, t)} = \frac{\sum _{a \ne b}{\hat{z}^{(i, t)}_{(a,b)}}}{\# \hat{z}^{(i, t)}_{(a,b)}}, \end{aligned}$$

where $\# \hat{z}^{(i, t)}_{(a,b)}$ is the number latent indicator variables (i.e. the number of domain pairs in species *i*) These are the maximum likelihood estimates of the model parameters, given the current expected value of the indicator variable on whether each pair is clustering or non-clustering.

*(3) Convergence check* Compute the value of the marginal log-likelihood of the model at iteration *t* ($\text{MLL}^{(i, t)}$) given the updated parameters estimates ${\hat{\phi }}^{(i, t)}$ and ${\hat{\lambda }}^{(i, t)}$:

$$\begin{aligned} \text{MLL}^{(i, t)} = \sum _{a \ne b}{\log \Big ({\hat{\phi }}^{(i, t)} \cdot \text{Exp}\Big (\text{dist}_{(a,b)}^{(i)} ; {\hat{\lambda }}^{(i, t)}\Big )} + (1 - {\hat{\phi }}^{(i, t)}) \cdot \text{U}\Big (\text{dist}_{(a,b)}^{(i)} ; 0, {L^{(i)}}/{2}\Big )\Big ) \end{aligned}$$

If the difference between the two consecutive values of $\text{MLL}^{(i,t)}$ is lower than a certain $\epsilon$ (here set to $10^{-16}$) i.e. $\text{MLL}^{(i,t)} - \text{MLL}^{(i,t-1)} < \epsilon$ stop iterating and take ${\hat{\phi }}^{(i, t)}, {\hat{\lambda }}^{(i, t)}$ as the final maximum likelihood estimates for $\phi ^{(i)}, \lambda ^{(i)}$. Otherwise, move to the next iteration.

#### Sampling of initial values

The EM algorithm is guaranteed to increase the complete data log-likelihood of the model, and consequently its marginal log likelihood, with every iteration. The complete data log-likelihood function, however, has multiple local maxima and the algorithm will converge to only one of them, which strictly depends on the value of the initial parameter guesses and does not necessarily correspond to the global solution to the maximisation problem. As the algorithm is completely deterministic, multiple runs with the same set of starting values will always converge to the same solution. A way to address this issue is by re-running the algorithm with different initial parameter guesses and taking the estimates that give the highest final marginal log-likelihood. Accordingly, the EM algorithm was repeated with 100 different combinations of initial guesses for $\lambda _i$ and $\phi _i$, obtained by combining ten samples for each parameter.

In the case of ${\hat{\lambda }}^{(i, 0)}$, an initial estimate was obtained with the method of moments assuming that all functionally interacting pairs were exponentially distributed, that corresponds to computing the reciprocal of the mean pairwise distance on each chromosome:

$$\begin{aligned} {\hat{\lambda }}^{(i, 0)} = \frac{\#\text{dist}_{(a,b)}^{(i)}}{\sum _{a \ne b}{\text{dist}_{(a,b)}^{(i)}}}, \end{aligned}$$

where $\#\text{dist}_{(a,b)}^{(i)}$ denotes the total number of pairwise distances in species *i*. Further guesses were then obtained by sampling ten logarithmically spaced values that ranged from three orders of magnitude below to three orders of magnitude above the value obtained with the method of moments. The initial guesses for the fraction of functionally interacting domains that were also clustering, ${\hat{\phi }}^{(i)}_0$, was obtained by sampling ten logarithmically spaced values between 0.001 and 0.1. This interval was chosen based on the *a priori* assumption, derived from visual analysis of the data, that the proportion of clustered pairs was going to be somewhere within this interval.

### Implementation and data visualisation

All above described analyses were implemented in a collection of scripts written in the programming language Python v3.6. The computational time required for the analysis of this extensive dataset was optimised by allocating the processing of individual assemblies to independent CPU cores working in parallel. Moreover, most numerical calculations were carried out using the library NumPy v.1.15.2 that allows for efficient vectorised processing of data. The import and handling of Genbank files and phylogenetic trees took advantage of the libraries Biopython v.1.72 [48] and ete3 v3.1.1 [49], respectively. Summary plots were generated with the libraries Matplotlib v.3.0.0 and seaborn 0.9.0. The scripts are available at https://github.com/selenocysteine/conserved-chromosomal-clusters.

## Supplementary Information

**Additional file 1**. Table S1 and Figure S1.

## Data Availability

The datasets analysed during the current study as referenced in the Methods section are available in the following public repositories. NCBI Genbank Genomes: https://ftp.ncbi.nlm.nih.gov/genomes/genbank/. Assembly metadata: https://ftp.ncbi.nlm.nih.gov/genomes/GENOME_REPORTS/prokaryotes.txt. Accession numbers of all 14,178 genomes analysed: https://doi.org/10.6084/m9.figshare.15035619.v1. Pfam domains: https://ftp.ebi.ac.uk/pub/databases/Pfam/releases/Pfam32.0/Pfam-A.hmm.gz. Pfam GO terms: http://geneontology.org/external2go/pfam2go. GO terms descriptions: http://purl.obolibrary.org/obo/go.obo. Taxonomic classifications: https://ftp.ncbi.nlm.nih.gov/pub/taxonomy/new_taxdump/new_taxdump.tar.gz

## Abbreviations

GO: Gene ontology
GOBP: Gene ontology biological process
EM algorithm: Expectation-maximisation algorithm

## Footnotes

1 We describe our steps in annotating these “Pfam domains” in Annotation with Pfam domain coding regions.

2 In the absence of knowledge of whether the pair is clustering or non-clustering.
