# Supplementary material for: Computational modelling of chromosomally clustering protein domains in bacteria
Source: BMC Bioinformatics. 2021 Dec 14;22:593. doi: 10.1186/s12859-021-04512-x (PMC8670047; doi:10.1186/s12859-021-04512-x)
Supplement: Supplementary file 1 — Additional file 1. Table S1 and Figure S1. [file 12859_2021_4512_MOESM1_ESM.pdf]

Computational modelling of chromosomally clustering protein domains in bacteria

July 22, 2021

Supporting information

Table S1: Housekeeping Genes List of housekeeping protein coding genes from Archaea and Bacteria.

Taken from [1], these protein coding genes are used to compute a phylogenetic tree of the dataset with bcgTree [2]. Profiles whose accession IDs start with TIGR are taken from the TIGRFAM database (`http://tigrfams.jcvi.org/cgi-bin/index.cgi`, [3]); profiles whose accession IDs start with PF are taken from the Pfam database (`https://pfam.xfam.org`, [4]).

Accession ID	Gene name	Protein description
TIGR00344	*alaS*	alanine-tRNA ligase
PF00750.15	*argS*	tRNA synthetases class I (R) arginyl-tRNA synthetase
TIGR00459	*aspS_bact*	aspartate-tRNA ligase
TIGR02729	*cgtA*	Obg family GTPase CgtA
TIGR00152	*coaE*	dephospho-CoA kinase
TIGR00435	*cysS*	cysteine-tRNA ligase
TIGR00362	*dnaA*	chromosomal replication initiator protein DnaA
TIGR01391	*dnaG*	DNA primase
TIGR02350	*dnaK*	chaperone protein DnaK
TIGR00663	*dnaN*	DNA polymerase III, beta subunit
TIGR02397	*dnaX_nterm*	DNA polymerase III, subunit gamma and tau
TIGR03594	*engA*	ribosome-associated GTPase EngA
TIGR00436	*era*	GTP-binding protein Era
TIGR00959	*ffh*	signal recognition particle protein
TIGR00460	*fmt*	methionyl-tRNA formyltransferase
TIGR00496	*frr*	ribosome recycling factor
TIGR00064	*ftsY*	signal recognition particle-docking protein FtsY
TIGR00388	*glyS*	glycine-tRNA ligase alpha subunit
TIGR00389	*glyS_dimeric*	glycine-tRNA ligase
TIGR03263	*gmk*	guanylate kinase
PF01025.15	*grpE*	co-chaperone GrpE
TIGR01063	*gyrA*	DNA gyrase, A subunit
TIGR01059	*gyrB*	DNA gyrase, B subunit
TIGR00442	*hisS*	histidine-tRNA ligase
TIGR00392	*ileS*	isoleucine-tRNA ligase
TIGR00487	*infB*	translation initiation factor IF-2
TIGR00168	*infC*	translation initiation factor IF-3
TIGR00755	*ksgA*	ribosomal RNA small subunit methyltransferase A
TIGR01393	*lepA*	elongation factor 4
TIGR00396	*leuS_bact*	leucine-tRNA ligase
TIGR00575	*ligA*	DNA ligase NAD-dependent
TIGR00420	*mnmA*	tRNA (5-methylaminomethyl-2-thiouridylate)-methyltransferase
PF01795.15	*mraW*	MraW methylase family
TIGR01953	*nusA*	transcription termination factor NusA
TIGR00922	*nusG*	transcription termination/antitermination factor NusG

PF00162.15	*pgk*	phosphoglycerate kinase
TIGR00468	*pheS*	phenylalanine-tRNA ligase, alpha subunit
TIGR00471	*pheT_arch*	phenylalanine-tRNA ligase, beta subunit
TIGR00472	*pheT_bact*	phenylalanine-tRNA ligase, beta subunit
TIGR00019	*prfA*	peptide chain release factor 1
TIGR00408	*proS_fam_I*	proline-tRNA ligase
TIGR00409	*proS_fam_II*	proline-tRNA ligase
TIGR00337	*pyrG*	CTP synthase
TIGR00082	*rbfA*	ribosome-binding factor A
TIGR02012	*recA*	protein RecA
TIGR02191	*rnc*	ribonuclease III
TIGR00060	*rpiR*	ribosomal protein L18
TIGR01169	*rplA_bact*	ribosomal protein L1
TIGR01171	*rplB_bact*	ribosomal protein L2
PF00297.18	*rplC*	ribosomal protein L3
PF00573.18	*rplD*	ribosomal protein L4/L1 family
PF00281.15	*rplE*	ribosomal protein L5
PF00347.19	*rplF*	ribosomal protein L6
TIGR00158	*rplI*	ribosomal protein L9
PF00466.16	*rplJ*	ribosomal protein L10
TIGR01632	*rplK*	ribosomal protein L11
TIGR00855	*rplK*	ribosomal protein L7/L12
TIGR01066	*rplM_bact*	ribosomal protein L13
TIGR01067	*rplN_bact*	ribosomal protein L14
TIGR01071	*rplO_bact*	ribosomal protein L15
TIGR01164	*rplP_bact*	ribosomal protein L16
TIGR00059	*rplQ*	ribosomal protein L17
TIGR01024	*rplS_bact*	ribosomal protein L19
TIGR01032	*rplT_bact*	ribosomal protein L20
TIGR00061	*rplU*	ribosomal protein L21
TIGR01044	*rplV_bact*	ribosomal protein L22
PF00276.16	*rplW*	ribosomal protein L23
TIGR01079	*rplX_bact*	ribosomal protein L24
TIGR00062	*rpmA*	ribosomal protein L27
TIGR00009	*rpmB*	ribosomal protein L28
TIGR00012	*rpmC*	ribosomal protein L29
TIGR01031	*rpmF_bact*	ribosomal protein L32
TIGR01030	*rpmH_bact*	ribosomal protein L34
TIGR00001	*rpmI_bact*	ribosomal protein L35
TIGR02027	*rpoA*	DNA-directed RNA polymerase, alpha subunit
TIGR02013	*rpoB*	DNA-directed RNA polymerase, beta subunit
TIGR02386	*rpoC_TIGR*	DNA-directed RNA polymerase, beta subunit
TIGR02387	*rpoC1_cyan*	DNA-directed RNA polymerase, gamma subunit
TIGR01011	*rpsB_bact*	ribosomal protein S2
TIGR01009	*rpsC_bact*	ribosomal protein S3
TIGR01017	*rpsD_bact*	ribosomal protein S4
TIGR01021	*rpsE_bact*	ribosomal protein S5
TIGR00166	*rpsF*	ribosomal protein S6
TIGR01029	*rpsG_bact*	ribosomal protein S7
PF00410.15	*rpsH*	ribosomal protein S8
PF00380.15	*rpsI*	ribosomal protein S9/S16
TIGR01049	*rpsJ_bact*	ribosomal protein S10
PF00411.15	*rpsK*	ribosomal protein S11
TIGR00981	*rpsL_bact*	ribosomal protein S12
PF00416.18	*rpsM*	ribosomal protein S13/S18
TIGR00952	*rpsO*	ribosomal protein S15
TIGR00002	*rpsP*	ribosomal protein S16
PF00366.16	*rpsQ*	ribosomal protein S17
TIGR00165	*rpsR*	ribosomal protein S18
TIGR01050	*rpsS_bact*	ribosomal protein S19
TIGR00029	*rpsT*	ribosomal protein S20
TIGR00963	*secA*	preprotein translocase SecA subunit
TIGR00964	*secE_bact*	preprotein translocase SecE subunit
TIGR00810	*secG*	preprotein translocase SecG subunit
TIGR00967	*secY*	preprotein translocase SecY subunit
TIGR00414	*serS*	serine-tRNA ligase
TIGR00086	*smpB*	SsrA-binding protein
TIGR00418	*thrS*	threonine-tRNA ligase
TIGR00115	*tig*	trigger factor

TIGR02432	*tilS*	tRNA(Ile)-lysidine synthetase
TIGR00116	*tsf*	translation elongation factor Ts
TIGR00234	*tyrS*	tyrosine-tRNA ligase
TIGR00631	*uvrb*	excinuclease ABC subunit B
TIGR00422	*valS*	valine-tRNA ligase
TIGR00043	*ybeY*	probable rRNA maturation factor YbeY
TIGR00092	*ychF*	GTP-binding protein YchF

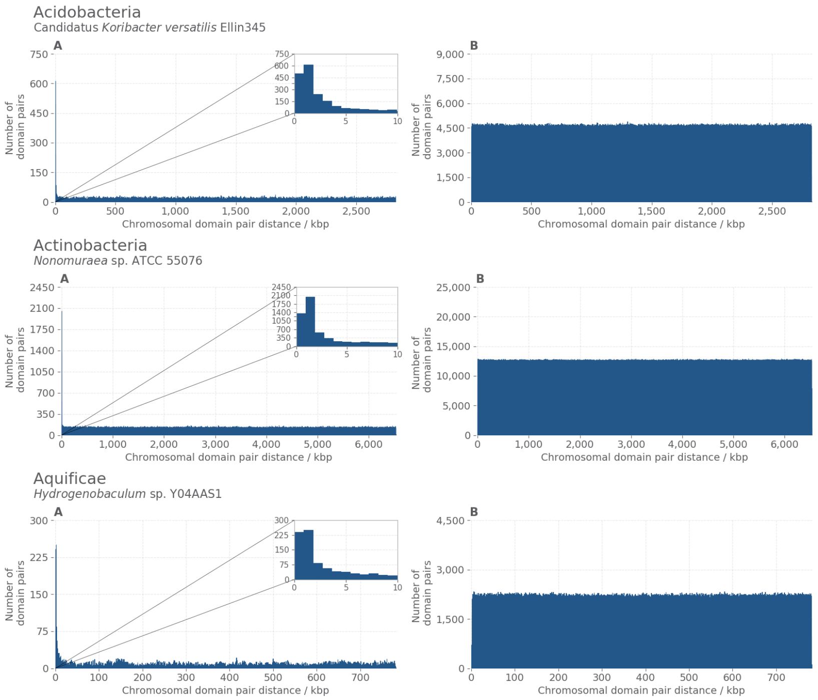

Figure S1: **Distributions of chromosomal distances for pairs of Pfam protein domain coding regions in different bacterial phyla.** Panels A: chromosomal distance, expressed in number of base pairs, between the two members of all pairs of domains where the two domains had one or more Gene Ontology Biological Process (GOBP) annotation in common (*functionally interacting pairs*). Inset on top right of each Panel A is a zoomed in view of the first 0 to 10 kbp to highlight the peak observed at low distances. Panels B: same distribution for pairs where the two domains did not have any common GOBP annotations (*other pairs*).

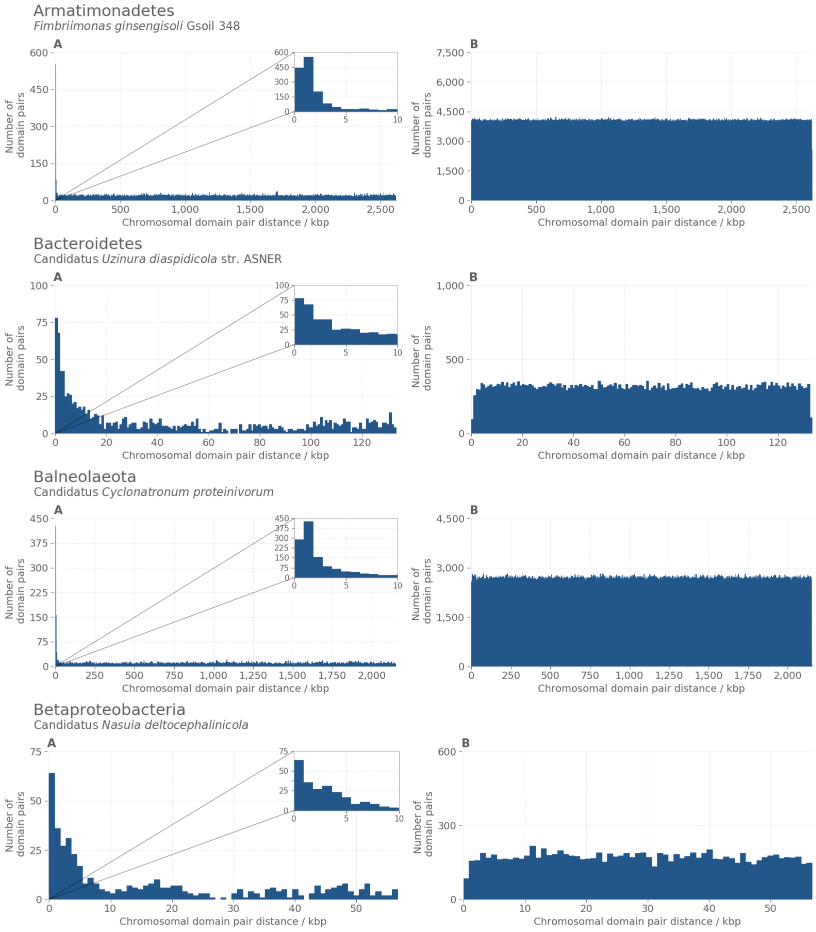
Armatimonadetes
Fimbriimonas ginsengisoli Gsoil 348
A
B
Number of domain pairs
Chromosomal domain pair distance / kbp
0 500 1,000 1,500 2,000 2,500
0 150 300 450 600
0 1,500 3,000 4,500 6,000 7,500
Bacteroidetes
Candidatus *Uzinura diaspidicola* str. ASNER
A
B
Number of domain pairs
Chromosomal domain pair distance / kbp
0 20 40 60 80 100 120
0 25 50 75 100
0 500 1,000
Balneolaeota
Candidatus *Cyclonatronum proteinivorum*
A
B
Number of domain pairs
Chromosomal domain pair distance / kbp
0 250 500 750 1,000 1,250 1,500 1,750 2,000
0 75 150 225 300 375 450
0 1,500 3,000 4,500
Betaproteobacteria
Candidatus *Nasuia deltocephalinicola*
A
B
Number of domain pairs
Chromosomal domain pair distance / kbp
0 10 20 30 40 50
0 25 50 75
0 300 600

Figure S1

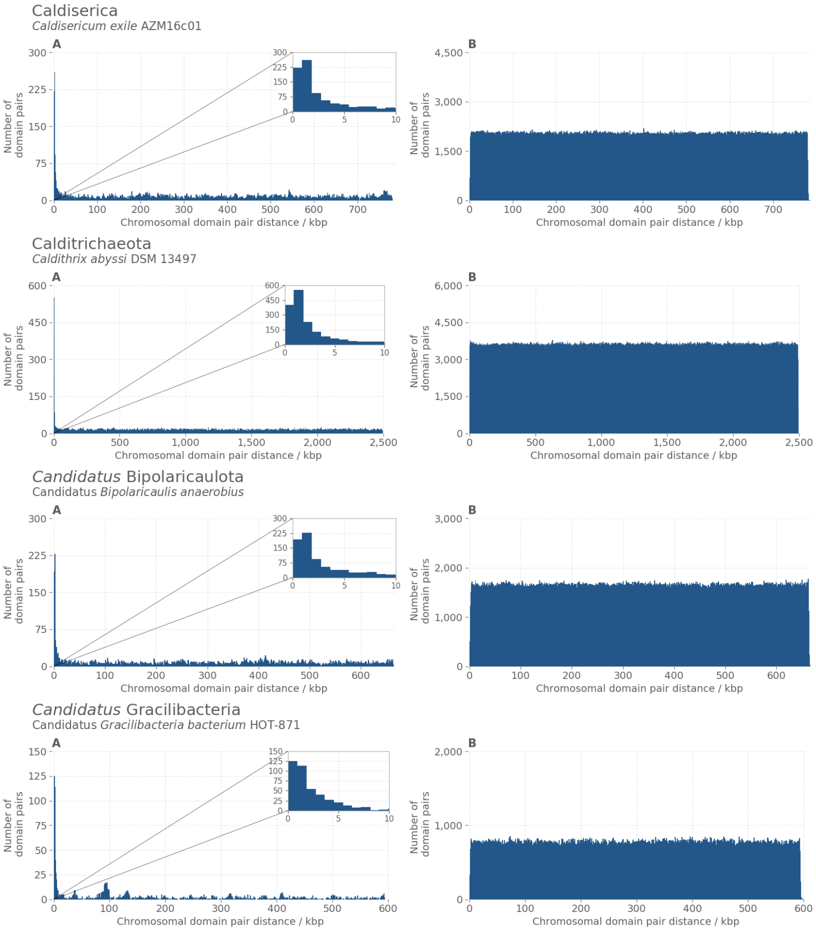
Caldiserica
Caldisericum exile AZM16c01
A
B
Number of domain pairs
Chromosomal domain pair distance / kbp
Calditrichaeota
Caldithrix abyssi DSM 13497
A
B
Number of domain pairs
Chromosomal domain pair distance / kbp
Candidatus Bipolaricaulota
Candidatus Bipolaricaulis anaerobius
A
B
Number of domain pairs
Chromosomal domain pair distance / kbp
Candidatus Gracilibacteria
Candidatus Gracilibacteria bacterium HOT-871
A
B
Number of domain pairs
Chromosomal domain pair distance / kbp

Figure S1

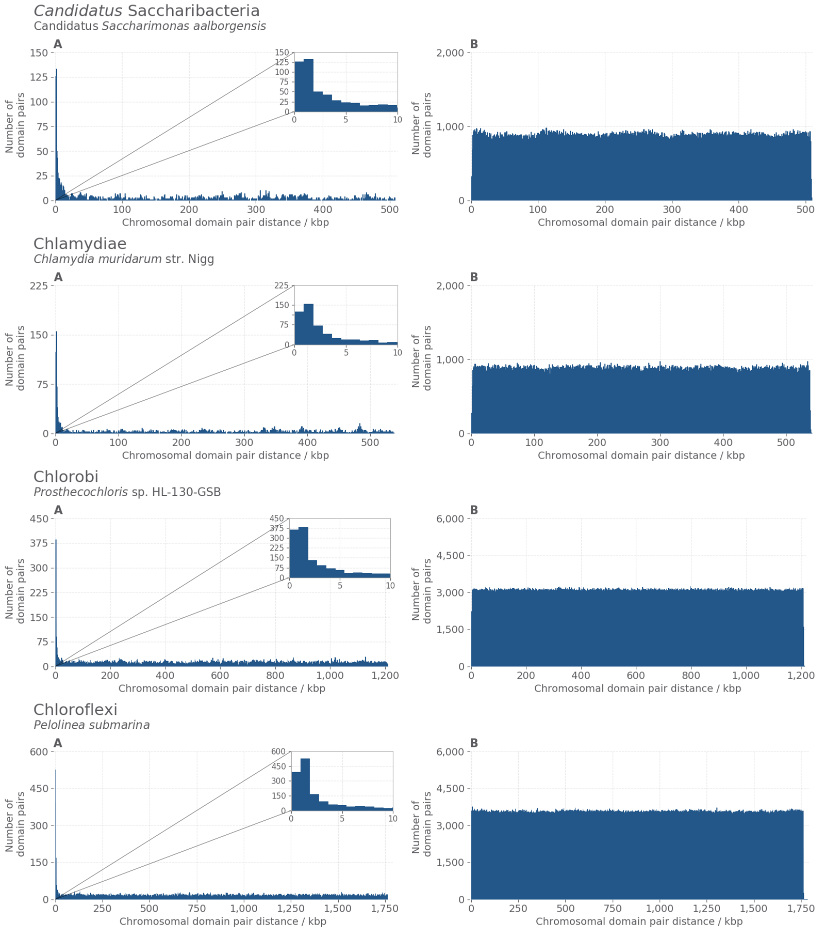

Candidatus Saccharibacteria
Candidatus *Saccharimonas aalborgensis*
A
B
Chlamydiae
Chlamydia muridarum str. Nigg
A
B
Chlorobi
Prosthecochloris sp. HL-130-GSB
A
B
Chloroflexi
Pelolinea submarina
A
B
Number of domain pairs
Chromosomal domain pair distance / kbp

Figure S1

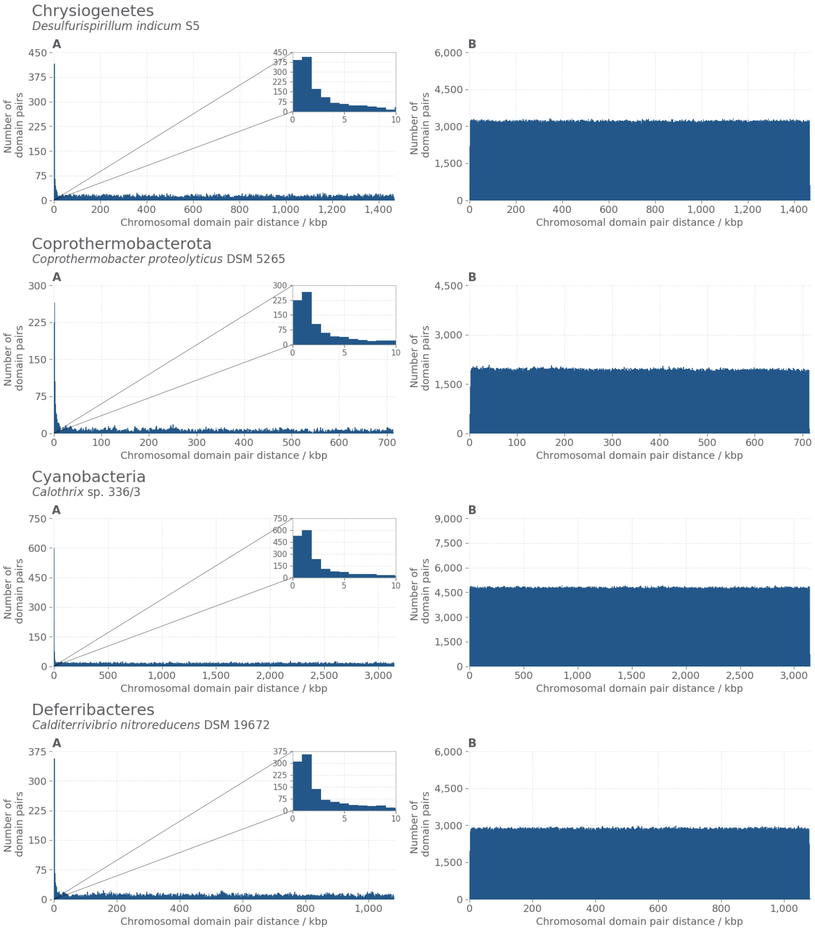
Chrysiogenetes
Desulfurispirillum indicum S5
A
B
Number of domain pairs
Chromosomal domain pair distance / kbp
Coprothermobacterota
Coprothermobacter proteolyticus DSM 5265
A
B
Number of domain pairs
Chromosomal domain pair distance / kbp
Cyanobacteria
Calothrix sp. 336/3
A
B
Number of domain pairs
Chromosomal domain pair distance / kbp
Deferribacteres
Calditerrivibrio nitroreducens DSM 19672
A
B
Number of domain pairs
Chromosomal domain pair distance / kbp

Figure S1

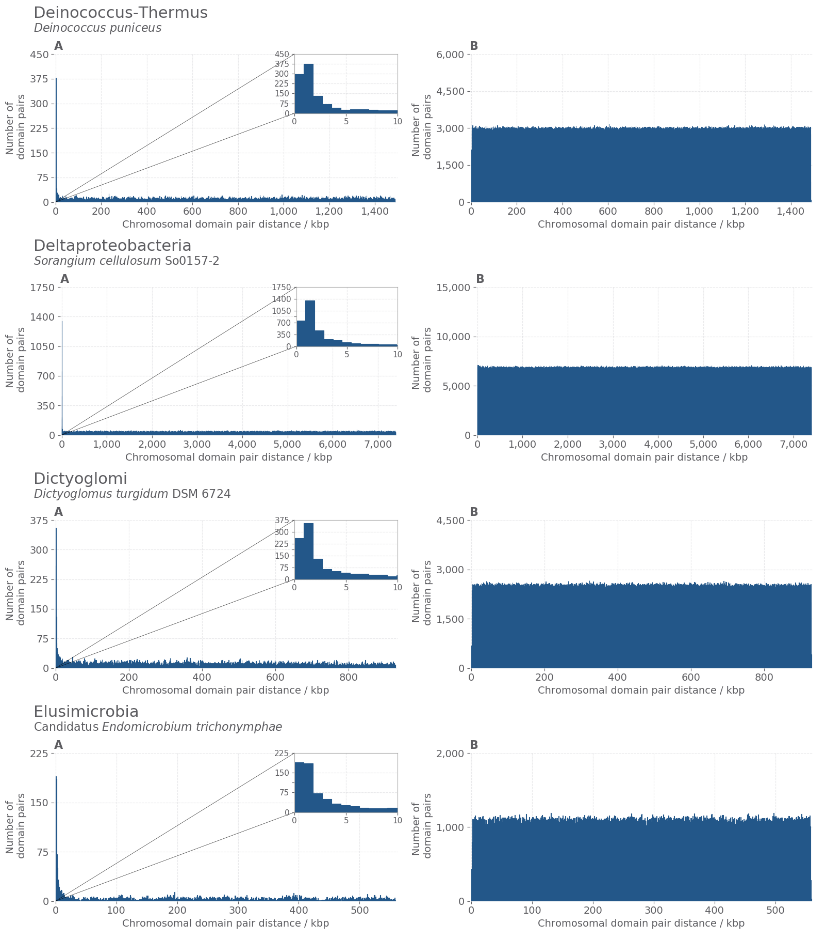

Deinococcus-Thermus
Deinococcus puniceus
A
B
Deltaproteobacteria
Sorangium cellulosum So0157-2
A
B
Dictyoglomi
Dictyoglomus turgidum DSM 6724
A
B
Elusimicrobia
Candidatus *Endomicrobium trichonymphae*
A
B
Number of domain pairs
Chromosomal domain pair distance / kbp

Figure S1

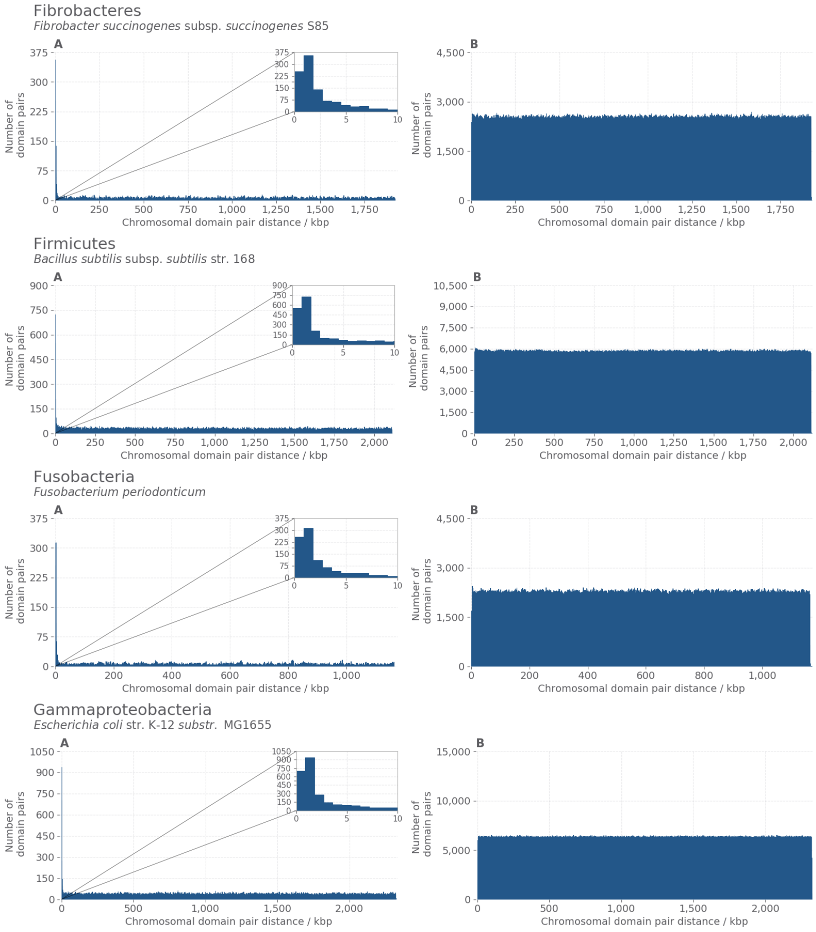
Fibrobacteres
Fibrobacter succinogenes subsp. succinogenes S85
A
B
Number of domain pairs
Chromosomal domain pair distance / kbp
Firmicutes
Bacillus subtilis subsp. subtilis str. 168
A
B
Number of domain pairs
Chromosomal domain pair distance / kbp
Fusobacteria
Fusobacterium periodonticum
A
B
Number of domain pairs
Chromosomal domain pair distance / kbp
Gammaproteobacteria
Escherichia coli str. K-12 substr. MG1655
A
B
Number of domain pairs
Chromosomal domain pair distance / kbp

Figure S1

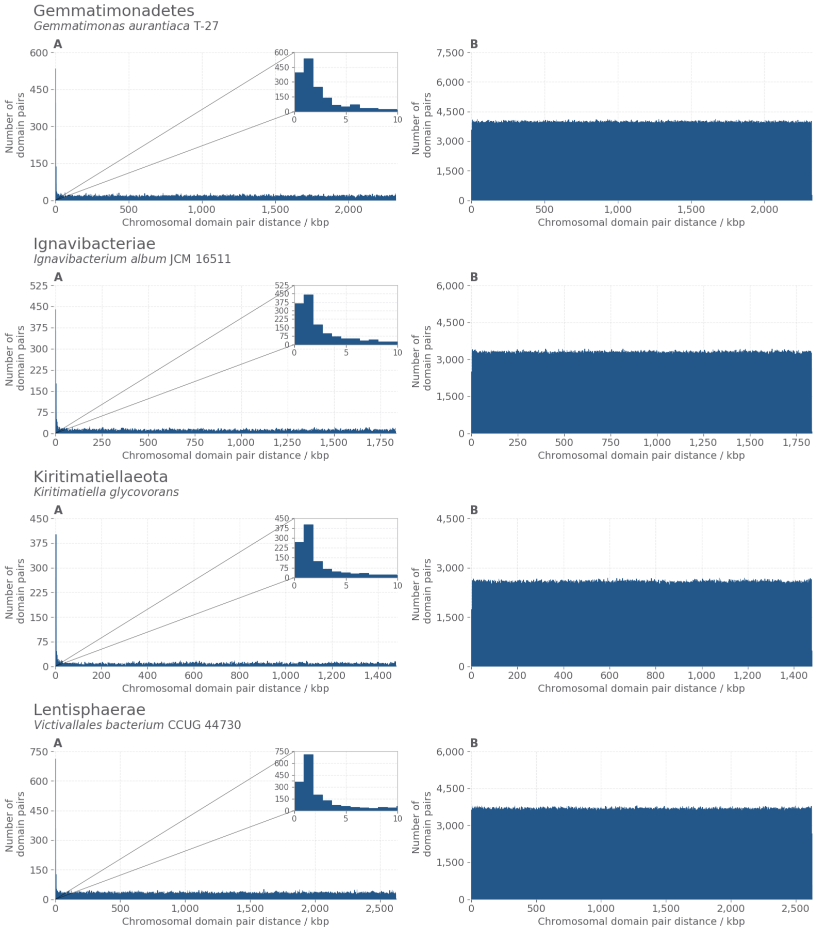
Gemmatimonadetes
Gemmatimonas aurantiaca T-27
A
B
Number of domain pairs
Chromosomal domain pair distance / kbp
Ignavibacteriae
Ignavibacterium album JCM 16511
A
B
Number of domain pairs
Chromosomal domain pair distance / kbp
Kiritimatiellaeota
Kiritimatiella glycovorans
A
B
Number of domain pairs
Chromosomal domain pair distance / kbp
Lentisphaerae
Victivallales bacterium CCUG 44730
A
B
Number of domain pairs
Chromosomal domain pair distance / kbp

Figure S1

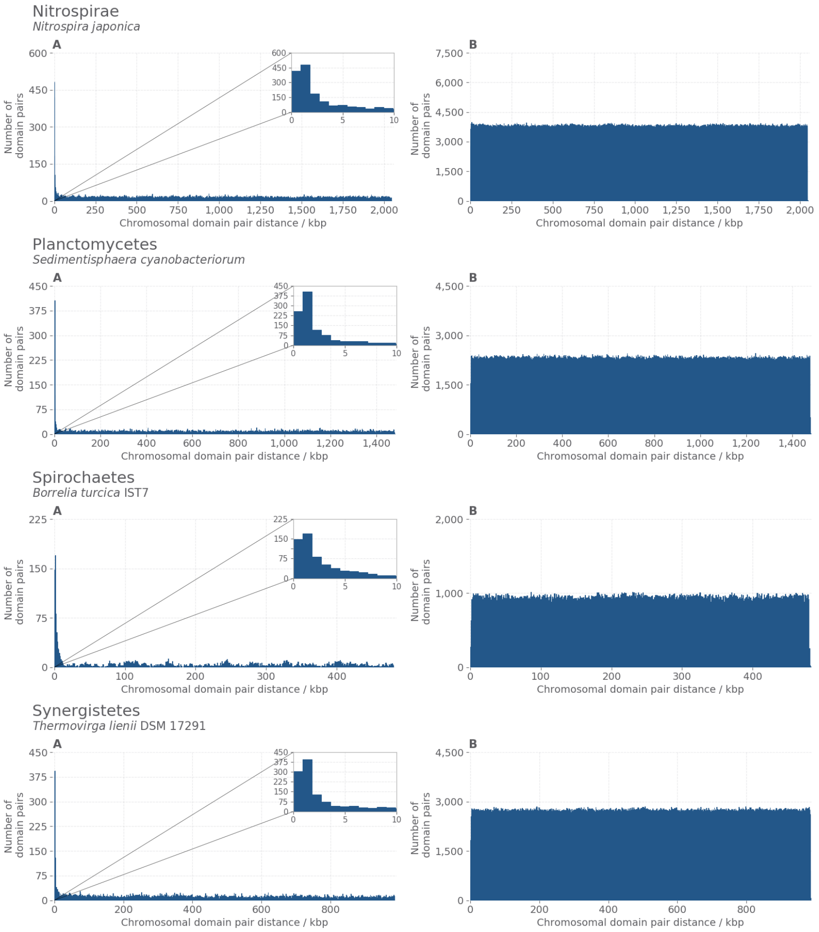
Nitrospirae
Nitrospira japonica
A
B
Number of domain pairs
Chromosomal domain pair distance / kbp
Planctomycetes
Sedimentisphaera cyanobacteriorum
A
B
Number of domain pairs
Chromosomal domain pair distance / kbp
Spirochaetes
Borrelia turcica IST7
A
B
Number of domain pairs
Chromosomal domain pair distance / kbp
Synergistetes
Thermovirga lienii DSM 17291
A
B
Number of domain pairs
Chromosomal domain pair distance / kbp

Figure S1

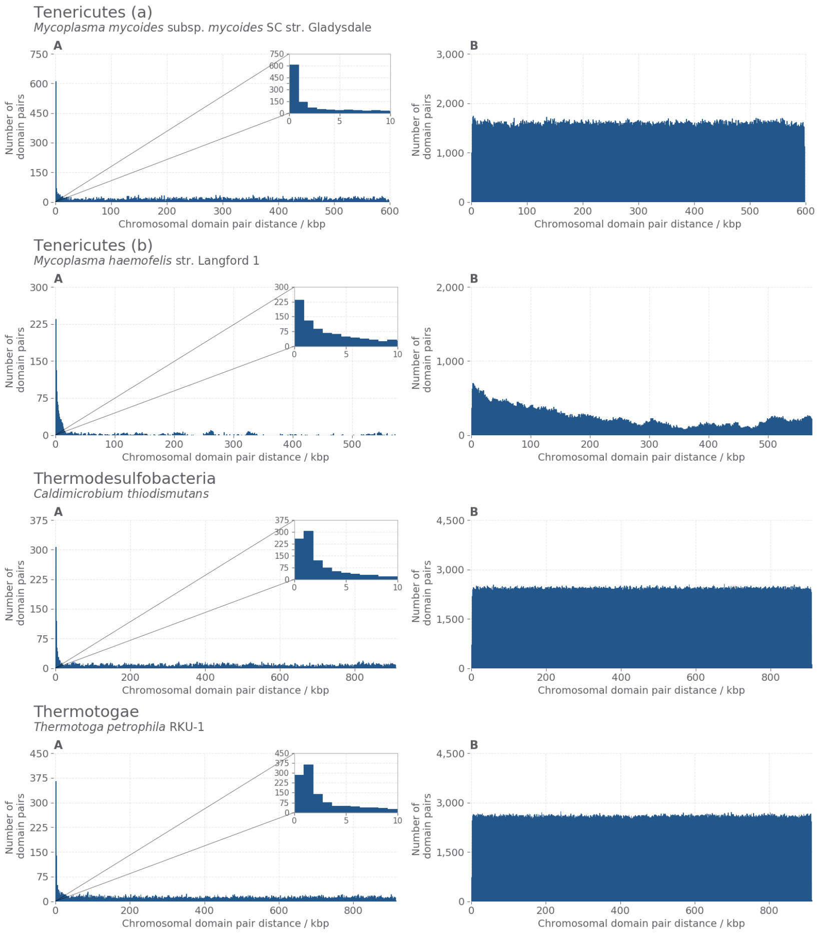
Tenericutes (a)
Mycoplasma mycoides subsp. mycoides SC str. Gladysdale
A
B
Number of domain pairs
Chromosomal domain pair distance / kbp
Tenericutes (b)
Mycoplasma haemofelis str. Langford 1
A
B
Number of domain pairs
Chromosomal domain pair distance / kbp
Thermodesulfobacteria
Caldimicrobium thiodismutans
A
B
Number of domain pairs
Chromosomal domain pair distance / kbp
Thermotogae
Thermotoga petrophila RKU-1
A
B
Number of domain pairs
Chromosomal domain pair distance / kbp

Figure S1

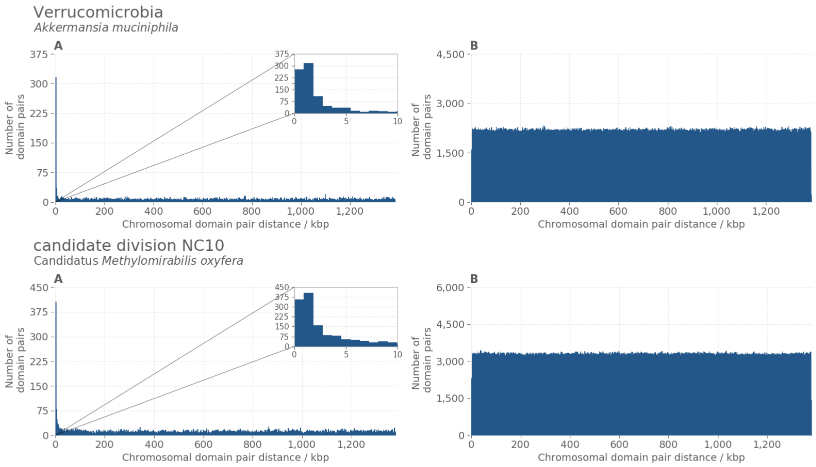

Figure S1

References

[1] Dupont, C.L., Rusch, D.B., Yooseph, S., Lombardo, M.J., Alexander Richter, R., Valas, R., Novotny, M., Yee-Greenbaum, J., Selengut, J.D., Haft, D.H., Halpern, A.L., Lasken, R.S., Nealson, K., Friedman, R., Craig Venter, J.: Genomic insights to SAR86, an abundant and uncultivated marine bacterial lineage. ISME Journal **6**(6), 1186–1199 (2012). doi:10.1038/ismej.2011.189

[2] Ankenbrand, M.J., Keller, A., Chain, F.: BcgTree: Automatized phylogenetic tree building from bacterial core genomes. Genome **59**(10), 783–791 (2016). doi:10.1139/gen-2015-0175

[3] Haft, D.H., Selengut, J.D., White, O.: The TIGRFAMs database of protein families. Nucleic Acids Research **31**(1), 371–373 (2003). doi:10.1093/nar/gkg128

[4] El-Gebali, S., Mistry, J., Bateman, A., Eddy, S.R., Luciani, A., Potter, S.C., Qureshi, M., Richardson, L.J., Salazar, G.A., Smart, A., Sonnhammer, E.L.L., Hirsh, L., Paladin, L., Piovesan, D., Tosatto, S.C.E., Finn, R.D.: The Pfam protein families database in 2019. Nucleic Acids Research **47**(D1), 427–432 (2019). doi:10.1093/nar/gky995
